# CT radiomics showed no improvement beyond volume dynamics for early lesion-level size response to immunotherapy in metastatic melanoma

**DOI:** 10.1186/s40644-026-01135-4

**Published:** 2026-10-01

**Authors:** Seval Akbal, Johan Constantin Schepers, Paul V. Op gen Oorth, Vincent Bleymehl, Moritz Schroth, Felix Winneknecht, Devayani Machiraju, Julius C. Holzschuh, Kevin S. Zhang, Sebastian Wohlfeil, Isabelle Ayx, Stefan O. Schoenberg, Heinz-Peter Schlemmer, Jessica C. Hassel, Lukas T. Rotkopf

**Affiliations:** 1 Department of Radiology, German Cancer Research Center (DKFZ), Im Neuenheimer Feld 280, 69120 Heidelberg, Germany; 2 Faculty of Medicine, Ruprecht-Karls-University of Heidelberg, Heidelberg, Germany; 3 Medical Faculty Mannheim, Ruprecht-Karls-University of Heidelberg, Mannheim, Germany; 4 Department of Dermatology, National Center for Tumor Diseases (NCT), University Hospital Heidelberg, Ruprecht-Karls-University of Heidelberg, Heidelberg, Germany; 5 Department of Dermatology, Venereology and Allergology, University Medical Center Mannheim, Ruprecht-Karls-University of Heidelberg, Mannheim, Germany; 6 Skin Cancer Unit, German Cancer Research Center (DKFZ), Heidelberg, Germany; 7 DKFZ Hector Cancer Institute at the University Medical Center Mannheim, Mannheim, Germany; 8 Department of Radiology and Nuclear Medicine, University Medical Center Mannheim, Ruprecht-Karls-University of Heidelberg, Mannheim, Germany; 9 Institute for Artificial Intelligence in Medicine, University Hospital Essen, Essen, Germany

**Keywords:** Metastatic melanoma, Immune checkpoint inhibitors, Radiomics, Tumor volumetry, Treatment response prediction

## Abstract

**Background:**

We evaluated whether CT intensity and texture radiomics provide predictive value beyond simple volume dynamics for lesion-level response prediction in metastatic melanoma treated with immune checkpoint inhibitors.

**Methods:**

We conducted a retrospective multicenter study including 158 patients (1,626 lesions) from an internal cohort (*n* = 129 patients with standardized protocols) and an external cohort (*n* = 29 patients with heterogeneous protocols). The primary endpoint was binary lesion-level size response (clinical benefit vs. progressive disease) at the second follow-up. We trained and compared Random Forest classifiers to predict this endpoint using clinical variables, volumetric features, radiomics, and combined predictors derived from baseline and first follow-up imaging. Models were trained on the internal cohort using patient-grouped cross-validation and applied unchanged to the external cohort. Paired differences in AUC were assessed against a ± 0.05 equivalence margin.

**Results:**

Baseline-only prediction of first follow-up response was near chance for all models. For predicting response at second follow-up, the volumetric model achieved an internal AUC of 0.84 [95% CI 0.78–0.88] and the radiomics model 0.86 [0.81–0.90]. In the external cohort, with 12 progression events, the volumetric and radiomics models achieved AUCs of 0.68 [0.50–0.85] and 0.68 [0.51–0.88], with no significant difference between any models. Organ-specific analysis showed no significant improvement from adding radiomics for any lesion site. Across all 288 radiomic features, the median absolute correlation with lesion volume was 0.37. Sensitivity analyses contained small or borderline gains that did not replicate externally.

**Conclusions:**

Volumetric features capture the primary predictive information for early lesion-level response, and CT radiomics showed no conclusive improvement over volume dynamics. Confirmation in larger independent cohorts is required before radiomic signatures can be recommended for this task.

**Clinical trial number:**

Not applicable.

**Supplementary information:**

The online version contains supplementary material available at https://doi.org/10.1186/s40644-026-01135-4.

## Background

Immune checkpoint inhibitors have transformed the treatment of metastatic melanoma, with combination regimens achieving median overall survival exceeding five years and durable responses in approximately half of treated patients [1, 2]. Despite these advances, a substantial proportion of patients do not respond to immunotherapy or experience significant immune-related adverse events, emphasizing the need for biomarkers that allow prediction of treatment response early in the therapeutic course to avoid futile treatment attempts. Current response assessment relies on standardized criteria that quantify changes in tumor burden through serial imaging. While the adapted immune response evaluation criteria in solid tumors (iRECIST) accounts for immunotherapy-specific response patterns such as pseudoprogression, it remains fundamentally dependent on size changes observed at follow-up imaging [3]. Predictive biomarkers derived from baseline or early follow-up imaging could support earlier identification of non-responders and guide timely treatment adjustment.

Radiomics has emerged as a promising approach for extracting quantitative imaging biomarkers beyond conventional size measurements. By quantifying texture, shape, and intensity distribution within tumor regions, radiomic features may capture biological characteristics relevant to treatment response [4, 5]. Several studies have demonstrated associations between radiomic signatures and immunotherapy outcomes in melanoma, with reported discrimination ranging from moderate to good in internal validation cohorts [6–8]. However, the clinical translation of radiomics faces a fundamental challenge: radiomic features show substantial sensitivity to imaging acquisition parameters, reconstruction algorithms, and scanner characteristics [9, 10]. Phantom studies have demonstrated that inter-scanner variability in feature values can approach or exceed the biological variability observed between patients [11]. Various harmonization strategies have been proposed, including statistical normalization and feature selection based on reproducibility testing; however, it remains uncertain whether these approaches contribute to preserving the predictive performance of radiomic features across heterogeneous clinical datasets [12, 13].

The majority of melanoma radiomics studies have been conducted within single institutions using standardized imaging protocols, limiting an assessment of their generalizability [11, 14, 15]. Notably, a recent multicenter study comparing radiomics to clinical models for checkpoint inhibitor response prediction from baseline imaging found that radiomics did not provide significant added value beyond established clinical parameters [16]. Hence, these findings raise questions regarding both fundamental limitations of radiomic approaches and their external validity and generalizability.

A related consideration is the relationship between radiomic features and tumor volume. Many texture and histogram-based features correlate strongly with lesion size, raising the possibility that radiomic models may primarily capture volume dynamics through indirect proxies rather than independent biological characteristics [17]. If volume change constitutes the dominant imaging signal for treatment response, as reflected in RECIST-based assessment, the incremental value of radiomics over simple volume dynamics warrants systematic evaluation.

This study addresses these gaps through a bicentric evaluation of early lesion-level response prediction in melanoma patients receiving immunotherapy. Lesion-level response was chosen because lesions of the same patient frequently respond differently under checkpoint inhibition, which matters for decisions about oligoprogression. Additionally, we examine whether radiomics provides differential benefit across organ-specific metastatic sites. Our primary hypothesis is that radiomics-containing models are equivalent to simple volume dynamics in the prediction of progression at second follow-up. The external and organ-specific comparisons are exploratory.

## Methods

### Study design and patient population

This retrospective bicentric study included two independent cohorts of patients with metastatic melanoma treated with immune checkpoint inhibitors. All procedures performed in this study were in accordance with the Declaration of Helsinki and relevant institutional guidelines. The study protocol was approved by the local ethics committees (ref. no. 2024–809 and S-415/2024). The requirement for written informed consent was waived. Patient privacy rights were observed. Both cohorts comprised consecutive patients imaged as part of clinical routine.

The internal cohort included patients from a single academic cancer center with standardized CT acquisition protocols between May 2013 and October 2023. The external cohort included patients from a collaborating institution with heterogeneous scanners and acquisition parameters reflecting routine clinical practice between March 2021 and July 2024.

Inclusion criteria for both cohorts were: (a) histologically confirmed stage IV melanoma at treatment initiation, (b) age of at least 18 years, (c) baseline contrast-enhanced CT including thoracic and abdominal coverage before treatment initiation, (d) at least one follow-up CT scan after baseline, and (e) at least one measurable metastatic lesion at baseline. Patients with insufficient image quality or incomplete clinical data were excluded. Concurrent malignancies were not an exclusion criterion.

### Image acquisition and lesion annotation

In the internal cohort, all CT examinations were performed using standardized protocols on one dual-energy CT scanner (Siemens SOMATOM Definition Flash, Siemens Healthineers, Erlangen, Germany). Contrast-enhanced imaging was acquired in either the arterial phase (thorax) or portal venous phase (abdomen) following intravenous administration of iodinated contrast agent. The external cohort was acquired across multiple scanners with varying acquisition protocols, reconstruction kernels, and contrast timing, reflecting typical heterogeneity encountered in multicenter settings. Acquisition and reconstruction parameters are summarized in Table S2.

**Fig. 1:**
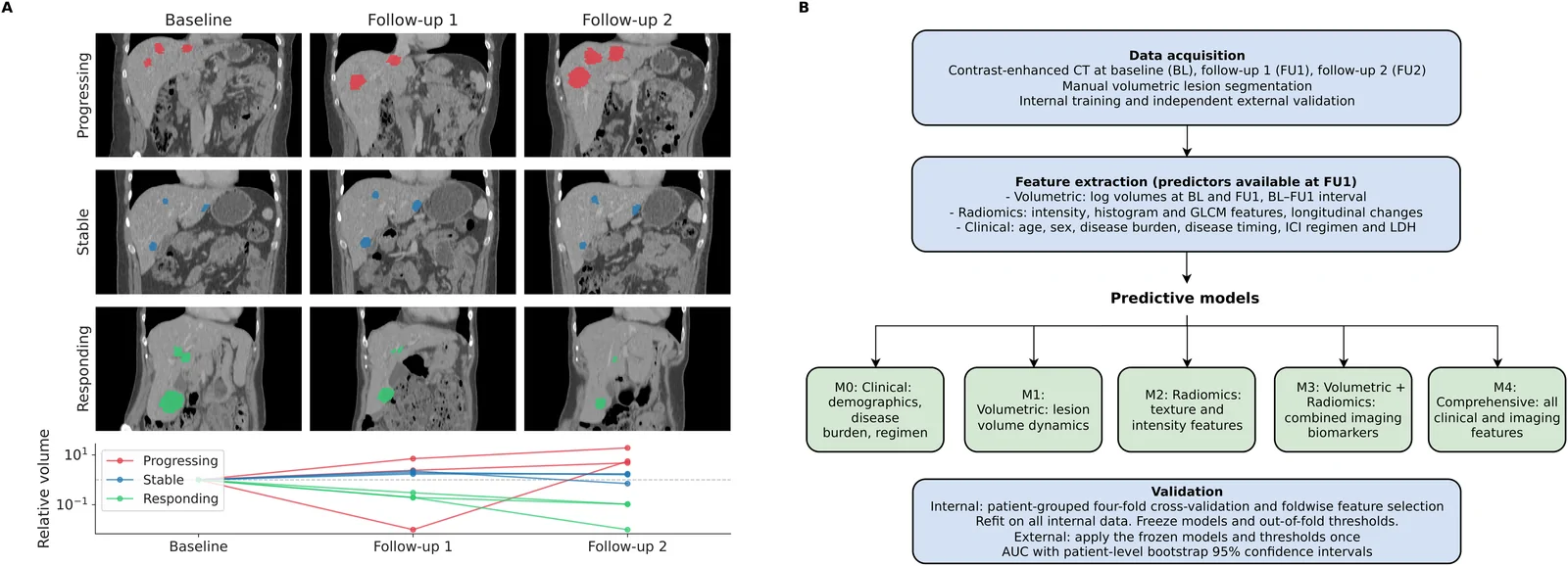
Visualization of the different lesion dynamics under therapy (**A**). Response is classified from lesion diameters, and volume is used only as a predictor. The analysis workflow (**B**) shows internal-cohort training and threshold selection, followed by a single application of the frozen models to the external cohort without a center indicator

Metastatic lesions were manually segmented by trained medical doctoral students using dedicated oncological imaging software (MintLesion, Mint Medical GmbH, Heidelberg, Germany). Up to the ten largest measurable lesions per lesion site per patient were annotated at baseline, with priority given to lesions with clearly delineable margins, and tracked longitudinally. All segmentations were reviewed by a board-certified radiologist, and all readers were blinded to response outcomes and the subsequent clinical course. Lesion-level response was classified from diameters using the RECIST 1.1 thresholds applied to the individual lesion: the long-axis diameter for all lesions except lymph nodes, which were assessed on the short axis. Progressive disease required an increase of at least 20% and at least 5 mm from the reference diameter (baseline at the first follow-up, the lesion-specific nadir at the second), partial response a decrease of at least 30% from baseline, and complete response disappearance of the lesion (volume 0 mL). All other lesions with complete measurements were classified as stable disease. New lesions and lesions with a missing measurement were not evaluable. Binary response was defined as clinical benefit (complete response, partial response, or stable disease) versus no clinical benefit (progressive disease) and is referred to as subsequent lesion-level size response. This lesion-level endpoint was evaluated at first and second follow-up. Lesion annotation and dynamics examples are shown in Fig. 1A and a flowchart of the analysis workflow in Fig. 1B.

### Feature extraction

Radiomic features were extracted in compliance with the Image Biomarker Standardization Initiative (IBSI) recommendations. Prior to feature calculation, images were resampled to isotropic 1 mm spacing. A fixed bin width of 5 Hounsfield units (HU) was applied in both cohorts for texture feature discretization, with intensity thresholds set between −1024 and 2048 HU. Feature extraction was performed using the radiomics toolbox integrated in MintLesion. The radiomic feature set included 72 features per lesion per timepoint, consisting of first-order histogram statistics, intensity-based features, and gray-level co-occurrence matrix (GLCM) texture features. For second follow-up prediction, delta (Δ) features were calculated as absolute or relative differences between first follow-up and baseline values. Volume parameters were log-transformed to reduce skewness. Clinical features included patient age, sex, treatment regimen, time since first diagnosis and since diagnosis of stage IV disease, the number of affected organs at baseline and first follow-up, and baseline lactate dehydrogenase (LDH). The cohort mean was used for the three internal patients without a measured LDH value.

### Model analysis

All models used Random Forest with recursive feature elimination (RFE) to ten features or less. For high-dimensional feature sets, variance filtering and univariate pre-selection to 50 features preceded RFE within each cross-validation fold [18]. Five nested predictor sets were compared: clinical (M0), volumetric (M1: log-transformed volumes at baseline and first follow-up and the baseline to first follow-up interval), radiomics (M2), volumetric plus radiomics (M3), and comprehensive (M4), as listed in Table S6. Performance in the internal cohort was evaluated using four-fold patient-grouped cross-validation stratified by outcome, with out-of-fold predictions aggregated for final metrics. The final model of each configuration was fitted on the full internal cohort and applied once, unchanged, to the external cohort. No model included a center indicator. The primary metric was area under the receiver operating characteristic curve (AUC), with 95% confidence intervals from cluster bootstrap resampling at the patient level. Sensitivity and specificity were reported at the Youden threshold of the internal out-of-fold predictions, which was applied unchanged to the external cohort. Precision-recall AUC, Brier score, balanced accuracy, and calibration intercept and slope are reported in Table S8. Model comparisons used paired differences in AUC with cluster bootstrap testing at the patient level against the volumetric model as reference, with Holm-Bonferroni correction for multiple comparisons within each cohort. Equivalence was concluded only when the entire confidence interval of the paired difference lay within a margin of ±0.05. Models were compared on the subset of lesions with complete feature data across all models. Spearman correlations with lesion volume were computed for all radiomic features. Within-patient dependence was addressed by patient grouping, patient-level resampling, and an equal-patient-weight sensitivity analysis. Adjusted *p*-values below 0.05 were considered statistically significant. All analyses were conducted using Python 3.12. We assessed study quality using the Radiomics Quality Score (RQS) [4], obtaining 18 of 36 points, with the item-level assessment provided in Supplementary Section S9 and Table S17.

## Results

### Study population and lesion characteristics

**Fig. 2:**
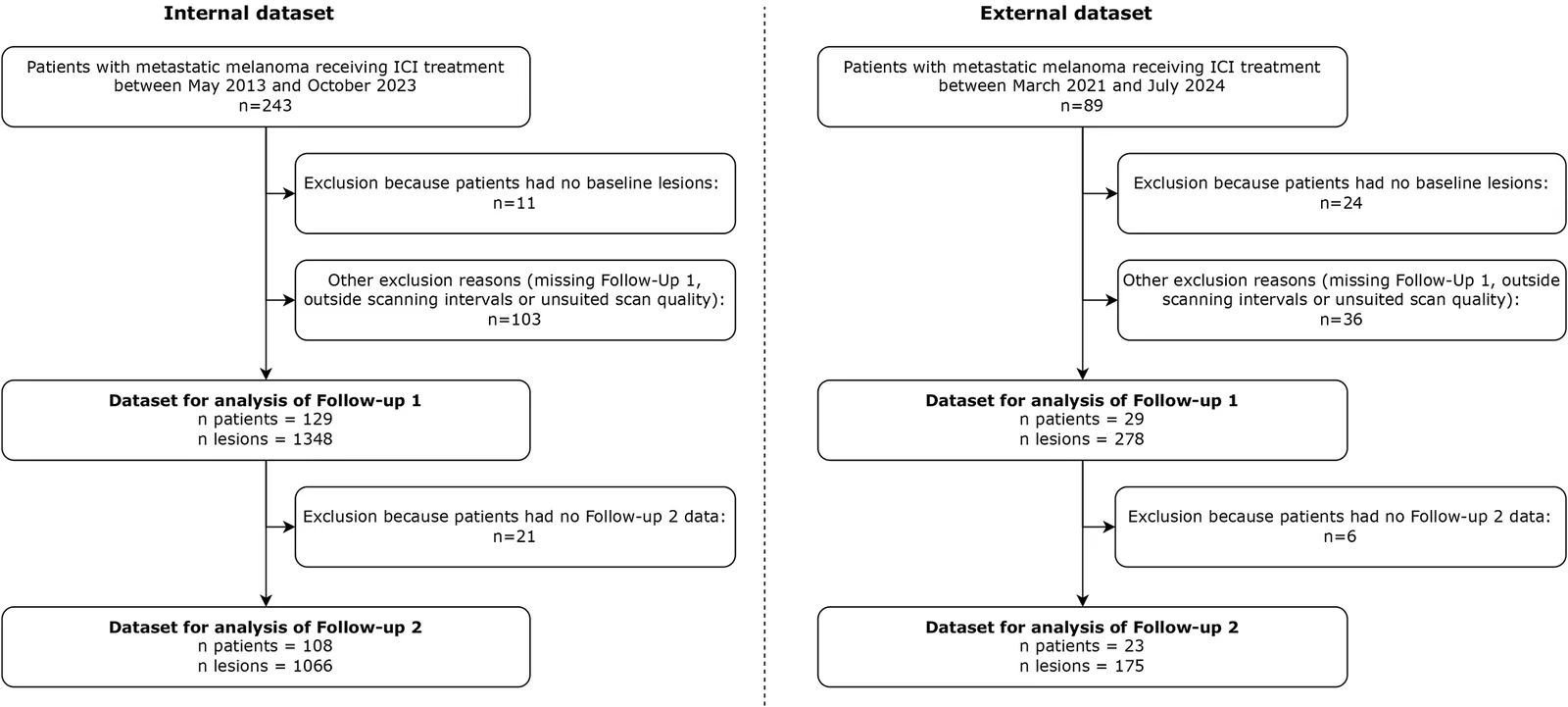
Flowchart showing the patient and lesion selection process for the internal and external datasets. FU1 and FU2 denote the first and second follow-up, respectively

The final study cohort consisted of 158 patients with 1,626 evaluable metastatic lesions from two centers. The primary second follow-up analysis included 131 patients with 1,241 lesions. The complete cohorts are described in Table 1. Patient demographics were comparable between centers regarding age and sex distribution. Treatment regimens differed substantially, with balanced representation across therapies in the internal cohort and predominant combination immunotherapy in the external cohort. Complete patient characteristics are presented in Table 1, formal between-cohort comparisons in Table S7, and the patient selection process is shown in Fig. 2.

**Table 1:** Characteristics of the 158 patients contributing lesions evaluable at the first follow-up

Parameter	Internal	External
Total, n	129	29
Age (years)	62.2 (12.6)	64.4 (13.4)
Sex, n (%)		
Male	60 (46.5)	18 (62.1)
Female	69 (53.5)	11 (37.9)
ICI regimen, n (%)		
Ipilimumab/nivolumab	51 (39.5)	26 (89.7)
Nivolumab	34 (26.4)	1 (3.4)
Pembrolizumab	44 (34.1)	1 (3.4)
Other	0 (0.0)	1 (3.4)
Baseline–FU1 interval (days)	95 (38, 174)	92 (51, 175)
FU1–FU2 interval (days)	89 (37, 618)	91 (12, 120)
Affected organs at baseline	2 (1, 10)	3 (1, 6)
LDH at baseline (U/L)	248 (134, 1451)	264 (146, 625)

Data are mean (SD) for age, median (min, max) for intervals, affected organs and LDH, and n (%) for categorical variables. Formal between-cohort comparisons are given in the supplementary cohort-comparison table

Lesion distribution varied between cohorts, with liver metastases being most frequent internally and lung metastases predominating externally. Median baseline lesion volumes were smaller in the external cohort. Detailed lesion characteristics are provided in Table 2.

**Table 2:** Characteristics of the included metastatic lesions evaluable at the first follow-up

Parameter	Internal	External
Total evaluable lesions, n	1348	278
Location, n (%)		
Liver	396 (29.4)	40 (14.4)
Lung	322 (23.9)	80 (28.8)
Lymph nodes	257 (19.1)	66 (23.7)
Skin	128 (9.5)	58 (20.9)
Other	245 (18.2)	34 (12.2)
Baseline volume (mL)	0.90 [0.32–2.96]	0.24 [0.06–1.70]
Follow-up 1 volume (mL)	0.94 [0.20–4.61]	0.23 [0.03–1.25]
Follow-up 2 volume (mL)	0.29 [0.00–2.06]	0.12 [0.00–0.89]
Baseline–FU1 interval (days)	92 (38, 174)	96 (51, 175)
FU1–FU2 interval (days)	85 (37, 618)	91 (12, 120)
Response at follow-up 1, n (%)		
Clinical benefit	991 (73.5)	243 (87.4)
Progressive disease	357 (26.5)	35 (12.6)
Response at follow-up 2, n (%)		
Clinical benefit	855 (80.2)	163 (93.1)
Progressive disease	211 (19.8)	12 (6.9)
Lesions evaluable at follow-up 2, n	1066	175

Data are median [IQR] for volumes, median (min, max) for intervals, and n (%) for categorical variables

### Predictions from baseline imaging

For prediction of response at first follow-up using only baseline features, all models demonstrated limited discriminative ability in both cohorts, as shown in Table S1, and no model exceeded the volumetric reference.

### Early response predictions

**Fig. 3:**
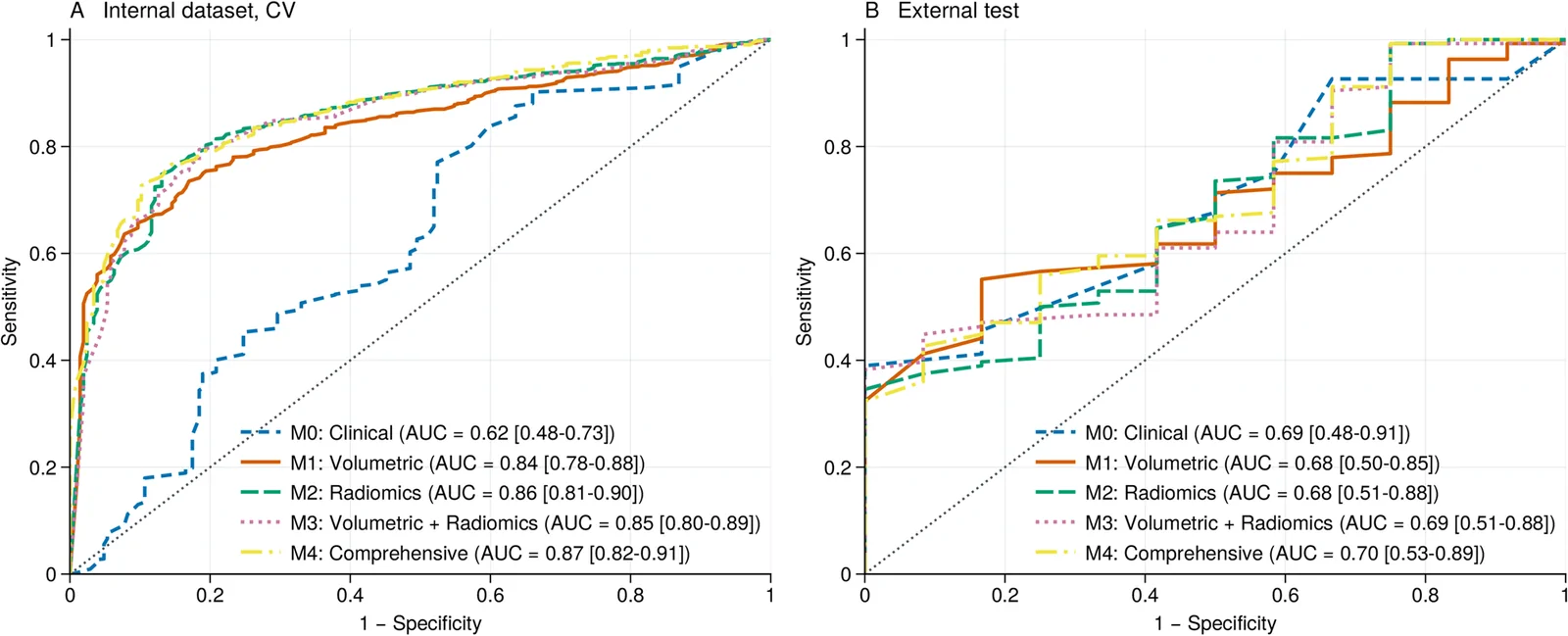
Receiver operating characteristic (ROC) curves comparing model performance for response prediction at second follow-up: internal patient-grouped cross-validation (**A**) and external test of the frozen models (**B**). Each curve is labeled with its AUC and 95% confidence interval

When early lesion dynamics from first follow-up became available as predictors, model performance improved substantially, as shown in Table 3 and Fig. 3. The volumetric model alone captured the majority of predictive signal from the volume dynamics. The radiomics model achieved comparable internal discrimination, and combining volumetric and radiomics features yielded a paired difference within the ±0.05 equivalence margin, as detailed in Table S9. The radiomics-only and comprehensive models showed small positive differences, which were not significant after Holm adjustment. The clinical model without imaging features demonstrated worse discrimination. In the external cohort, no model differed significantly from the volumetric model, and the confidence intervals were too wide to establish equivalence or a deficit. Precision-recall AUC exceeded the prevalence baseline internally, and calibration decreased externally for all models, as documented in Table S8 and Figure S1. Early volume change alone, without a fitted model, retained most of the discrimination of the volumetric model internally and exceeded it externally, as described in Section S3 of the supplement. Removing the lesions that had disappeared at the first follow-up lowered the AUC of all models alike without changing the paired differences, as demonstrated in Table S19. In an exploratory patient-level analysis of the internal cohort, adding radiomics to a patient-level volumetric model did not improve discrimination of a RECIST-style patient response, as shown in Table S18.

**Table 3:** Discrimination of the five models for lesion-level response at the second follow-up

Model	Internal AUC	Internal p	Internal sensitivity / specificity	External AUC	External p	External sensitivity / specificity
M0: Clinical	0.62 [0.48–0.73]	<0.001	0.77 / 0.48	0.69 [0.48–0.91]	1.000	0.93 / 0.33
M1: Volumetric	0.84 [0.78–0.88]	—	0.74 / 0.83	0.68 [0.50–0.85]	—	0.68 / 0.50
M2: Radiomics	0.86 [0.81–0.90]	0.456	0.75 / 0.87	0.68 [0.51–0.88]	1.000	0.61 / 0.58
M3: Volumetric + Radiomics	0.85 [0.80–0.89]	0.456	0.79 / 0.82	0.69 [0.51–0.88]	1.000	0.67 / 0.42
M4: Comprehensive	0.87 [0.82–0.91]	0.141	0.73 / 0.90	0.70 [0.53–0.89]	1.000	0.58 / 0.67

Intervals are patient-level cluster-bootstrap 95% confidence intervals. Adjusted *p*-values were calculated as cluster-bootstrap tests against M1

### Correlation between radiomic features and lesion volume

**Fig. 4:**
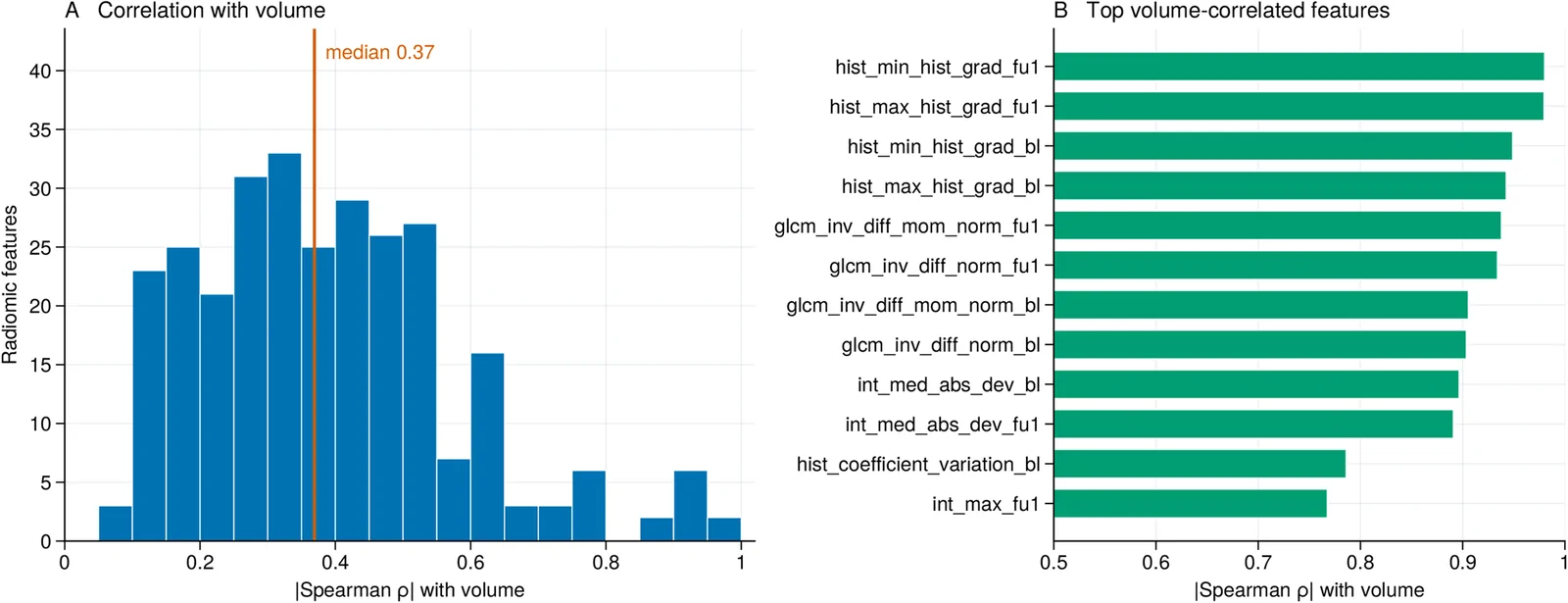
Spearman correlations between lesion volume and all 288 radiomic features of the internal cohort: distribution of the absolute correlation (**A**) and the most volume-correlated features (**B**) The feature names hist_min_hist_grad and hist_max_hist_grad denote the minimum and maximum gradients of the intensity histogram, respectively. The names glcm_inv_diff_mom_norm and glcm_inv_diff_norm denote the normalized inverse difference moment and normalized inverse difference of the gray-level co-occurrence matrix, respectively. The name int_med_abs_dev denotes the median absolute deviation of voxel intensities, hist_coefficient_variation denotes the coefficient of variation of the intensity histogram, and int_max denotes the maximum voxel intensity. The suffixes _bl and _fu1 indicate measurements at baseline and the first follow-up, respectively

To investigate potential volume confounding, we computed Spearman correlations between lesion volumes and all 288 radiomic features, as shown in Fig. 4. Across all 288 radiomic features, the median absolute Spearman correlation with lesion volume was 0.37. Of the 288 features, 19 (6.6%) showed strong correlation (|ρ| >0.7), and 8 (2.8%) exceeded 0.9, up to |ρ| = 0.98. Volume-residualized, volume-decorrelated, and redundancy-filtered radiomics retained their discrimination, and none improved on volume dynamics, as summarized in Table S11.

### Organ-specific subgroup analysis

Exploratory subgroup analysis by lesion site in the internal cohort compared volumetric against combined volumetric-radiomics models, as shown in Table 4. No organ subgroup demonstrated statistically significant improvement from adding radiomic features to volumetric features. All subgroups were underpowered. Across lesion-size subgroups, no group favored radiomics after adjustment, as shown in Table S12 and Figure S2.

**Table 4:** Exploratory comparison of the volumetric model (M1) against the combined volumetric and radiomics model (M3) by lesion site, for response at the second follow-up, restricted to the internal cohort

Lesion site	Lesions, n (patients)	Progression events, n	M1: Volumetric AUC	M3: Volumetric + Radiomics AUC	Adjusted p
Liver	299 (48)	71	0.83 [0.72–0.91]	0.85 [0.76–0.93]	1.000
Lung	262 (61)	8	0.71 [0.47–0.96]	0.71 [0.51–0.93]	1.000
Lymph nodes	207 (57)	47	0.62 [0.49–0.75]	0.70 [0.60–0.80]	1.000
Skin	105 (28)	22	0.62 [0.47–0.87]	0.75 [0.58–0.90]	0.715
Other	193 (48)	63	0.68 [0.55–0.80]	0.72 [0.61–0.82]	1.000

### Sensitivity analyses

Alternative learners and repeated cross-validation did not change the comparison, as reported in Section S6 of the supplement. ComBat harmonization did not improve the external discrimination of the radiomics model. Cross-center distribution filtering resulted in a numerically higher external radiomics AUC than volume dynamics without a significant difference. Internally, the distribution-filtered comprehensive model showed a gain that was significant after Holm adjustment but did not replicate externally, as detailed in Table S13. The extended feature set with higher-order texture families, spatial filters, and four bin widths did not exceed the volumetric reference after adjustment, as shown in Table S14. Retention at the second follow-up was associated with first follow-up response, and inverse-probability weighting left the model comparison unchanged, as documented in Table S15. Inter-observer agreement from an independent second reading was high for first-order and texture features. Volume and the maximal in-plane diameter, the analogue of the long-axis diameter that defines the endpoint, were both highly reproducible, whereas the maximal three-dimensional diameter, which the study does not use, was not, as demonstrated in Table S16.

## Discussion

This study found no conclusive improvement of CT radiomics over simple volume dynamics for early lesion-level response to immunotherapy, including in organ-specific subgroup analyses. Internally, only the clinical model exceeded the ±0.05 equivalence margin, while the radiomics-only and comprehensive models showed small positive differences within the equivalence margin. In the external cohort, no model differed from volume dynamics, but the estimates were too imprecise to establish equivalence or a deficit.

The comparable prediction performance of volumetric features and radiomics on internal validation, combined with the lacking incremental value of radiomics, suggests substantial redundancy between these feature sets. Correlation analysis revealed that only a minority of radiomic features were strongly associated with lesion volume, and radiomics retained their discrimination after residualization on volume, exclusion of volume-correlated features, and redundancy filtering. Volume confounding has been recognized as a methodological concern in radiomics research but is rarely addressed through direct comparison [10, 17].

Subgroup analysis explored whether radiomic features provided differential benefit across anatomical lesion sites and lesion sizes. After Holm-Bonferroni correction for multiple comparisons, no organ or size subgroup demonstrated statistically significant improvement from adding radiomics to volumetric features. All subgroups were underpowered. The largest lesions, in which texture can be calculated most stably, showed a numerical gain that did not survive adjustment, so noise-limited texture in small lesions cannot be excluded as a contributor to the negative result. Texture features nevertheless demonstrated high inter-rater stability in an independent second reading, which argues against contour noise as the sole explanation. This is consistent with prior evidence that volumetric measurements are robust to changes in reconstruction algorithms, whereas texture feature variability due to reconstruction kernels can exceed inter-reader variability and approach inter-patient biological variability [11, 19]. Reconstruction-dependent variation in radiomic features has also been reported in FDG PET [20].

Our results are in line with several previous studies. Peisen et al. for instance found no significant advantage of adding radiomics to a clinical baseline model in predicting early response or survival at 12 months from baseline CT imaging [21]. A study using PET-CT and blood biomarkers for differentiating pseudoprogression from true progression reported that a model combining blood markers and radiomics slightly outperformed a delta-volume model [22]. Strikingly, Ter Maat et al. showed that a deep learning model using a neural network trained on CT imaging lesions was not better than a pure clinical model in forecasting treatment outcome [23]. A study comparing baseline single- and dual-energy radiomic features in predicting response at first follow-up found no benefit of the single-energy features [24], which is in line with our findings based on single-energy CT image data. Regarding the prediction of early overall survival, research findings are more equivocal: one study reported that a four-feature signature including size features outperformed RECIST 1.1 [25], and a similar study demonstrated that the addition of delta-radiomics improved prediction of best response, overall and progression-free survival up to 12 months after baseline but did not test volume-based models [26]. Beyond the absence of a volume-dynamics comparator, earlier positive reports differ from this study in their single-center designs with standardized protocols, baseline-only signatures, patient-level endpoints, and small cohorts with limited validation. Our negative result regards only incremental value over volume dynamics for a size-based lesion-level endpoint and does not invalidate associations reported for other endpoints. Strong correlation of a minority of features with volume has also been described for PET radiomics [27].

Retrospective harmonization did not change the conclusion. ComBat did not improve external discrimination, and cross-center distribution filtering, which selects features with similar marginal distributions in both cohorts and may therefore reflect case mix as well as acquisition, resulted in a numerically higher external estimate without a significant difference. Neural network-based extraction methods were not tested by this study. PET-based lung cancer studies have also examined feature harmonization [28] and external validation of subtype classification [29].

This study has several limitations. First, the external cohort was relatively small, with only 29 patients, of whom 20 contributed 12 progression events, with an unequal distribution between lesions with and without clinical benefit. Consequently, the external confidence intervals are too wide to establish equivalence, and no external subgroup analyses were possible. Larger multicenter studies are required to confirm these findings.

Second, the treatment distribution differed substantially between cohorts, with combination immunotherapy predominating externally, and the cohorts also differed in lesion sites, lesion size, and outcome prevalence, as evident from Tables 1 and 2. Because regimen is closely associated with center, treatment-related differences in response patterns cannot be separated from acquisition differences. Thoracic and abdominal lesions were segmented on different reconstructions with different kernels and contrast phases.

Third, the lesion-level analysis treats observations from the same patient as partially independent, which may inflate performance estimates. We addressed this through patient-grouped cross-validation and patient-level cluster bootstrap resampling. Within-patient response concordance was low and equal patient weighting changed the AUCs of the imaging models only slightly. Fourth, the second follow-up results naturally include only surviving patients, biasing outcome estimations, although inverse-probability weighting left the comparison unchanged. Finally, the clinical model does not capture all established prognostic factors of melanoma.

## Conclusions

In this study, CT radiomics showed no conclusive improvement over volume dynamics for early lesion-level response prediction. Future studies should investigate whether alternative deep learning approaches can extract predictive information from imaging beyond what volume dynamics already provide.

## Electronic supplementary material

Below is the link to the electronic supplementary material.

Supplementary Material 1

## Data Availability

No datasets were generated or analysed during the current study.

## Abbreviations

AUC: Area under the receiver operating characteristic curve
CI: Confidence interval
CT: Computed tomography
GLCM: Gray-level co-occurrence matrix
HU: Hounsfield unit
IBSI: Image Biomarker Standardization Initiative
ICC: Intraclass correlation coefficient
ICI: Immune checkpoint inhibitor
IQR: Interquartile range
iRECIST: Immune Response Evaluation Criteria in Solid Tumors
LDH: Lactate dehydrogenase
PR-AUC: Area under the precision-recall curve
RECIST: Response Evaluation Criteria in Solid Tumours
RFE: Recursive feature elimination
ROC: Receiver operating characteristic
RQS: Radiomics Quality Score
SD: Standard deviation
