# Supplementary material for: CT radiomics showed no improvement beyond volume dynamics for early lesion-level size response to immunotherapy in metastatic melanoma
Source: Cancer Imaging. 2026 Oct 1;26:138. doi: 10.1186/s40644-026-01135-4 (PMC13632448; doi:10.1186/s40644-026-01135-4)
Supplement: Supplementary file 1 [file 40644_2026_1135_MOESM1_ESM.pdf]

Supplementary Material

S1. Supplementary methods

Image acquisition

Internal contrast-enhanced CT examinations used a Siemens SOMATOM Definition Flash, usually with arterial thoracic and portal venous abdominal series. Acquisition and reconstruction parameters are listed in Table S2. External parameters describe an illustrative subset of examinations.

Feature extraction

Trained readers segmented lesions in MintLesion, and a board-certified oncological radiologist reviewed all contours. Readers were blinded to response outcomes. Feature extraction used isotropic 1 mm voxels and a fixed bin width of 5 HU in both cohorts.

Each lesion contributed 72 features per timepoint. Intensity statistics describe raw CT attenuation, histograms describe gray-level distributions, and gray-level co-occurrence matrix (GLCM) features describe relationships between neighboring voxels. Shape features were excluded from the primary models (Table S3).

For first-follow-up response, radiomic predictors came from baseline imaging. For second-follow-up response, they included baseline and first-follow-up values and their absolute and relative changes. Second-follow-up imaging was not used as a predictor.

Lesion-level response

RECIST 1.1 size thresholds were applied to individual lesions. Measurements used the long axis, except for lymph nodes, which used the short axis. Complete response required disappearance, partial response a reduction of at least 30% from baseline, and progression an increase of at least 20% and 5 mm from the reference diameter. The reference was baseline at first follow-up and the smaller of the baseline and first-follow-up diameters at second follow-up. Other evaluable lesions were stable. Clinical benefit comprised complete response, partial response or stable disease, compared with progression. Lesions without an evaluable response were excluded for that timepoint (Section S7).

Models

Five models used clinical variables (M0), volume dynamics (M1), radiomics (M2), volume dynamics plus radiomics (M3), or all features (M4). For the primary second-follow-up analysis, M1 used log volumes at baseline and first follow-up and the interval between them. M0 included demographic and disease characteristics, treatment regimen and baseline lactate dehydrogenase (LDH). Three missing internal LDH values were replaced by the cohort mean. No center indicator was included. Table S6 lists the predictors for each endpoint.

Model fitting and external validation

Random forests used 200 trees, a maximum depth of 50, uniform class weights and seed 42. For larger feature sets, variance filtering at 0.01 and univariate screening to 50 features preceded recursive feature elimination to ten predictors. Selection and fitting were repeated within each training fold. Four-fold cross-validation was stratified by response and grouped by patient. Out-of-fold predictions provided the internal metrics and Youden operating thresholds. Selected features are reported in Table S10.

Final models were fitted on all internal data and applied to the external cohort without refitting or changing thresholds. All primary feature selection and model development used internal data alone. Section S6 describes exploratory analyses that also used external feature distributions.

Lesions that disappeared at first follow-up were retained with log volume log(0.01), zero first-follow-up radiomic values, and changes calculated from those zeros. Both cohorts used this encoding. The exclusion analysis reused predictions without refitting (Table S19).

Statistical analysis

We estimated 95% confidence intervals using 2000 patient-level cluster bootstrap resamples, retaining all lesions of each sampled patient. Models were compared on shared complete cases using paired AUC differences from the volumetric reference and two-sided bootstrap p-values. Holm–Bonferroni correction was applied within each comparison family. Internal equivalence required the entire confidence interval to lie within ± 0.05 AUC. Secondary metrics included progression-class precision–recall AUC, Brier score, calibration and operating characteristics.

Software

Extraction settings and software versions are listed in Table S4. All analyses used Python.

S2. Cohort comparability

Age and sex did not differ significantly between cohorts. Treatment regimen, lesion site, baseline volume and response rates differed (Table S7). Lesion-level comparisons are descriptive and treat lesions as independent. Radiomic feature distributions also varied between cohorts (Table S5).

S3. Model performance

Baseline models had limited discrimination in both cohorts (Table S1). These analyses used 1342 lesions from 128 internal patients and 255 lesions from 26 external patients, including 355 and 33 progression events, respectively.

In the primary analysis, no radiomics-containing model significantly exceeded volume dynamics after multiplicity adjustment. Only the combined model met the internal equivalence criterion. External intervals were too wide to establish equivalence (Tables S8 and S9). Calibration was poorer externally. Internal decision curves showed little separation between the imaging models (Figure S1).

Radiomics restricted to raw intensity features also did not outperform M1 externally (AUC 0.70 versus 0.68). An unfitted score based on early log-volume change matched M1 internally (AUC 0.84) and had a higher external point estimate (0.83 versus 0.68).

Excluding lesions that disappeared at first follow-up lowered discrimination. Internal paired differences changed little, while external estimates shifted toward the volumetric model without a significant difference (Table S19).

S4. Radiomics and lesion volume

Most features had weak or moderate correlations with volume (Figure 4, main text). We repeated the models after removing linear volume effects, excluding features correlated with volume ($|\rho| > 0.5$), or dropping one feature from highly correlated pairs ($|\rho| > 0.9$). The variants retained discrimination, and none significantly outperformed volume dynamics (Table S11).

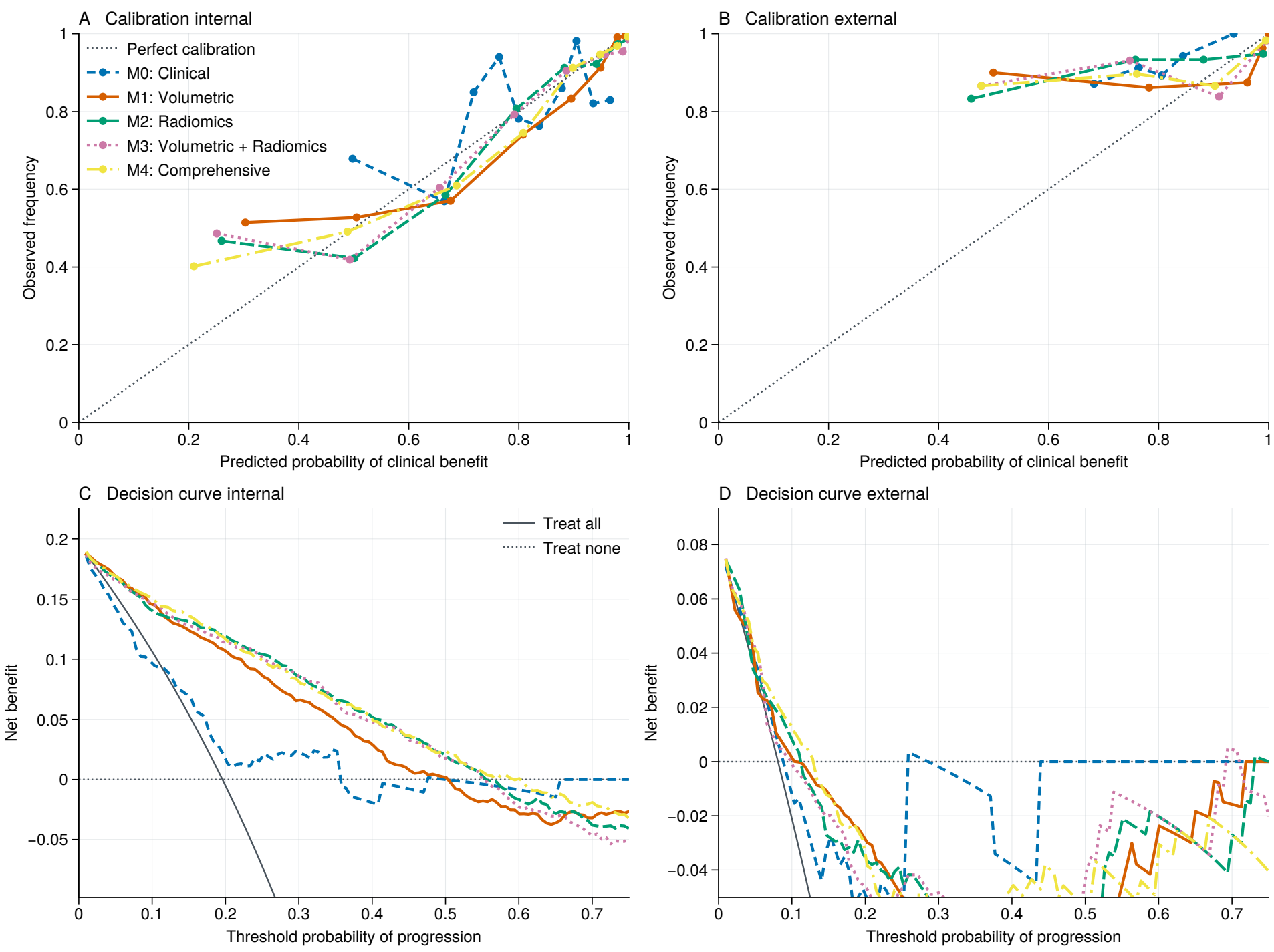

Figure S1: Calibration and decision curves.

S5. Subgroup analyses

Exploratory analyses compared M1 and M3 within organ and baseline-volume subgroups of the internal cohort, using patient-grouped cross-validation. Confidence intervals were unadjusted and p-values were Holm-corrected across subgroups. No subgroup difference remained significant after adjustment, including a nominal gain among the largest lesions (Table 4, main text, Table S12 and Figure S2). Wide intervals limit conclusions about subgroup effects.

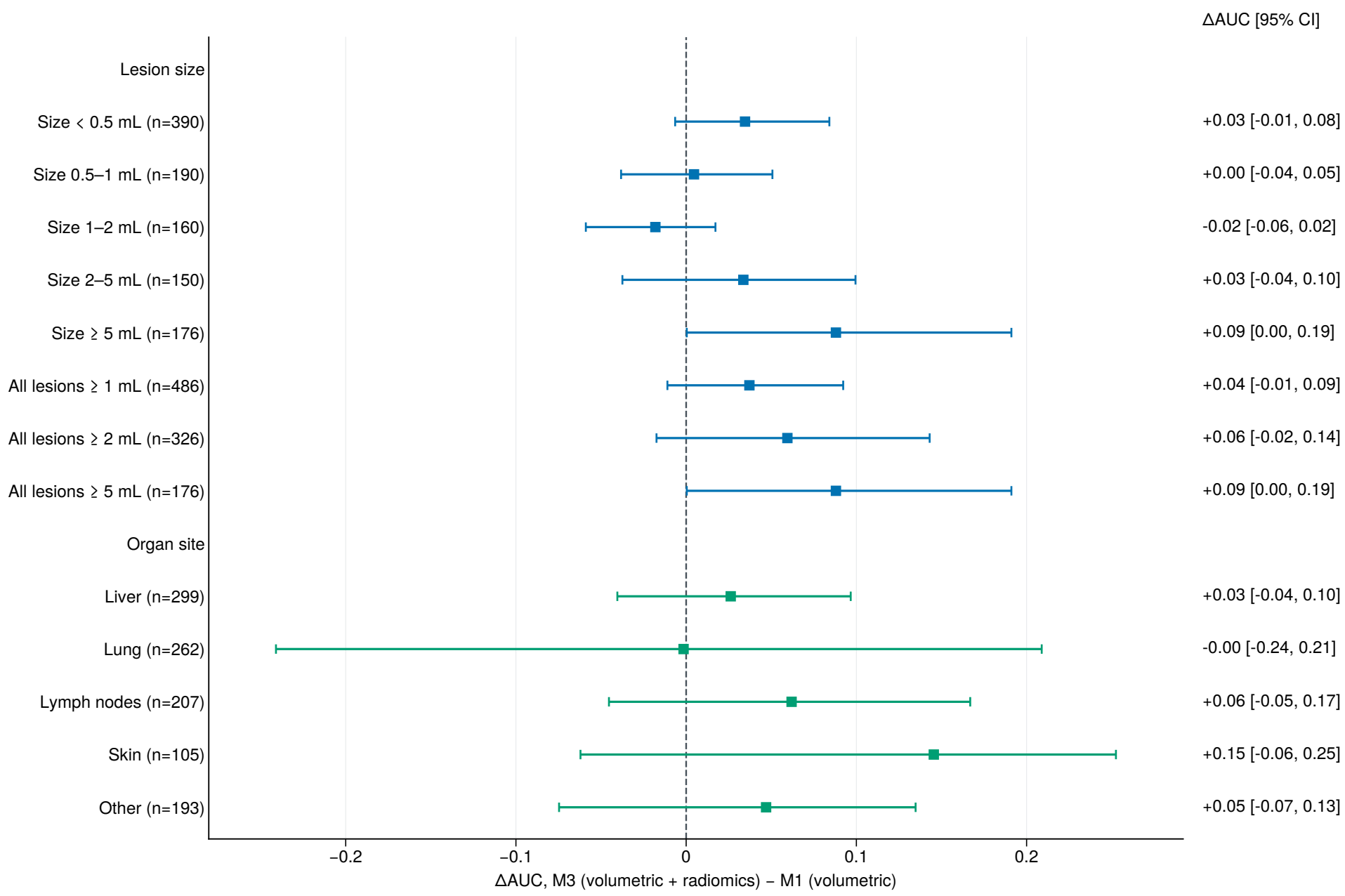

Figure S2: Incremental discrimination by lesion size and organ.

S6. Robustness analyses

Logistic regression, support vector machines and gradient boosting gave the same overall result using the same folds and feature selection. AUC differences from adding radiomics ranged from −0.019 to +0.015, with all intervals including zero. Across ten random patient-grouped splits, the average difference between M3 and M1 was +0.040. These repetitions used the same patients and do not provide independent confirmation.

ComBat adjusted for center while preserving log baseline volume. Distribution filtering retained features with small between-cohort differences: standardized mean difference $\leq$0.5 and Kolmogorov–Smirnov p $\geq$ 0.01. Both procedures used feature distributions from both cohorts without using outcomes, so they are not independent external tests. Feature differences can reflect patient characteristics as well as scanning protocols. ComBat did not improve external discrimination. Filtering gave a higher external radiomics estimate without a significant gain. It improved the comprehensive model internally after adjustment, but that gain did not replicate externally (Table S13).

An extended PyRadiomics analysis included higher-order textures, shape, wavelet and Laplacian-of-Gaussian features at bin widths of 5, 10, 25 and 64 HU. Sixteen configurations were compared with M1 under identical cross-validation. Some unadjusted differences favored extended features, but none remained significant after correction (Table S14, Figure S3). These sensitivity analyses used PyRadiomics in place of the primary MintLesion extraction.

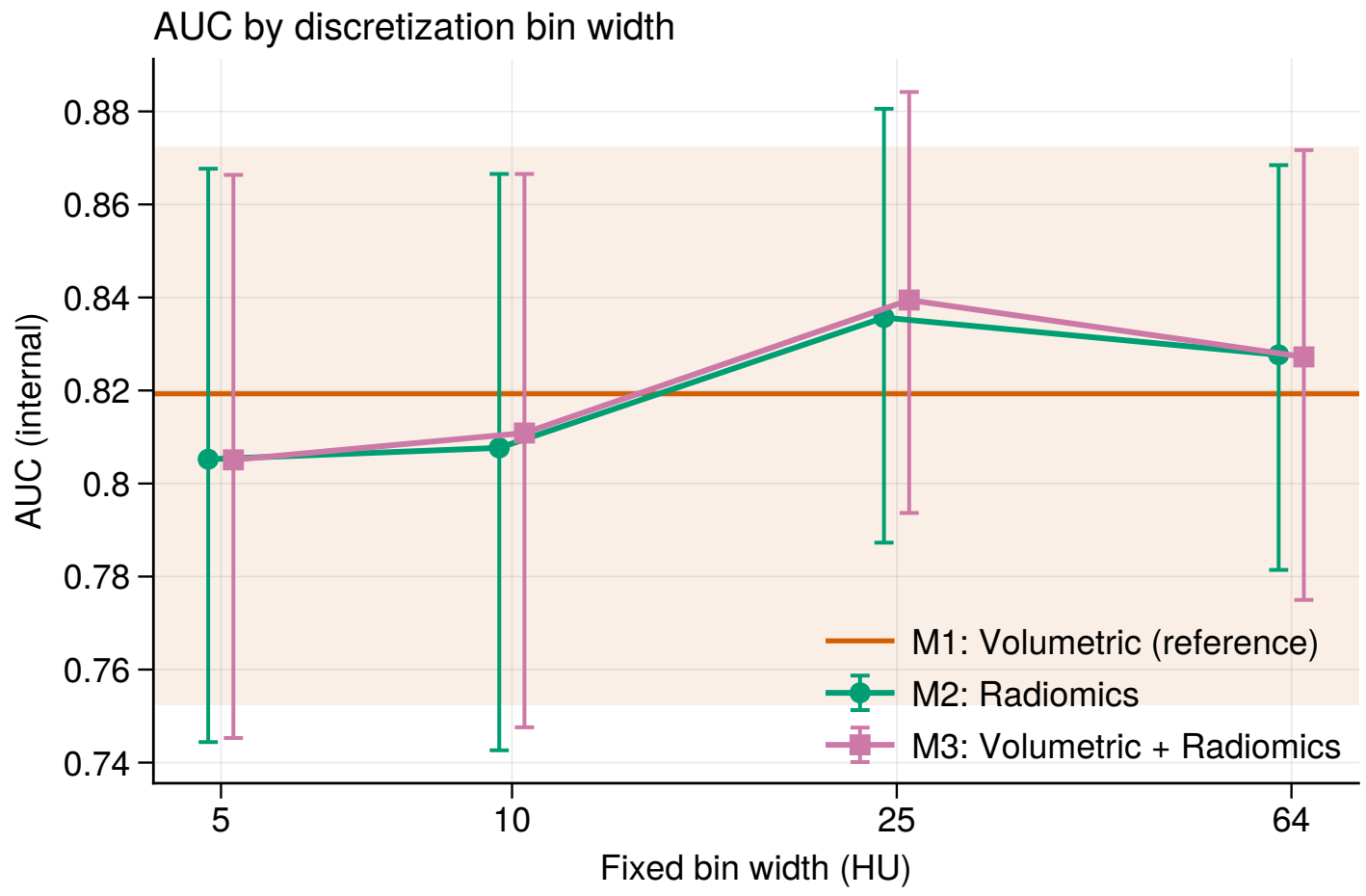

Figure S3: Discrimination by discretization bin width.

S7. Follow-up attrition

Most losses at second follow-up reflected a missing scan. Lesions lost to follow-up were more likely to have progressed at first follow-up (Table S15). We modeled patient retention by logistic regression using age, disease burden, first-follow-up response and the baseline-to-first-follow-up interval. Retained patients received inverse-probability weights, with probabilities bounded below at 0.05. Weighting changed imaging-model AUCs by at most 0.013 and left the model comparison unchanged. Results still apply only to lesions with continued imaging.

S8. Segmentation and software reproducibility

We compared independently drawn baseline contours from seven internal patients, matched by lesion overlap. Each contour pair was processed on the same image with identical PyRadiomics settings at

25 and 64 HU. ICC(2,1) agreement was high for first-order and texture features (Table S16, Figure S4). Agreement was also high for volume and in-plane diameters, but poor for the three-dimensional diameter, which was not used for response assessment. Some contours were drawn on different reconstructions. Differences therefore reflect both the reader and the images used for contouring. This small baseline study does not establish reproducibility of follow-up contours or change features.

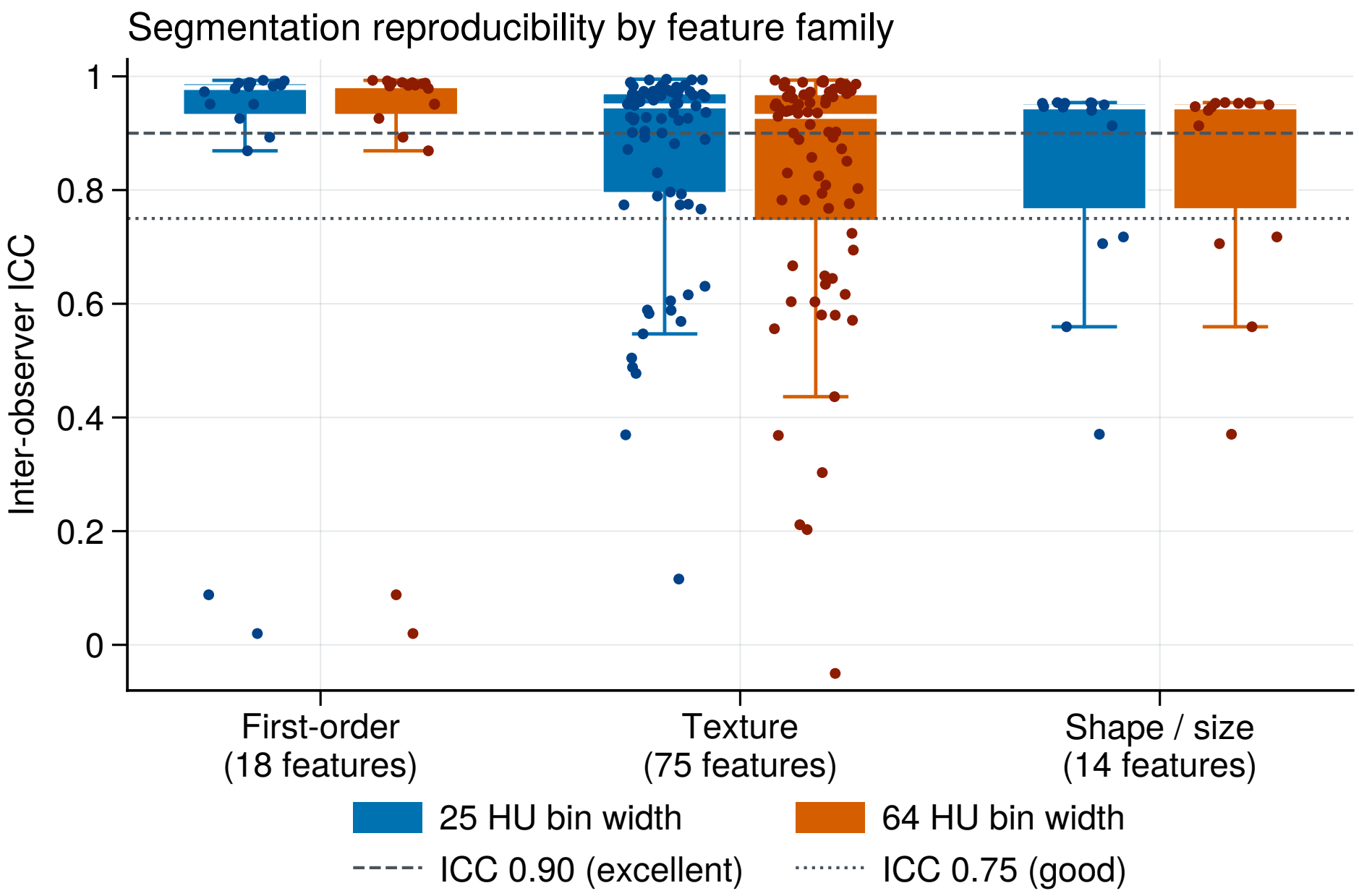

Figure S4: Inter-observer feature reproducibility.

For 37 matched feature definitions at 5 HU, MintLesion and PyRadiomics had high rank correlations: median Spearman ρ was 0.96 for intensity and 0.93 for GLCM features. Absolute values nevertheless differed between packages. The primary models used MintLesion.

S9. Radiomics Quality Score

The self-assessed Radiomics Quality Score was 18 of 36 (Table S17). Code and de-identified feature matrices are available on request.

S10. Patient-level sensitivity analysis

An exploratory internal-cohort analysis used RECIST-style patient response at second follow-up, based on the sum of longest diameters. P0 used clinical variables. P1 used log total volumes at baseline and first follow-up, percentage changes in total volume and the sum of diameters, lesion count and scan interval. P2 used per-patient means of radiomic features, and P3 combined P1 and P2. Random forests used feature selection within each fold, retaining up to eight features, and five-fold stratified cross-validation. Radiomics did not improve on P1 after Holm adjustment (Table S18). This remains exploratory because formal patient-level rules for target lesions and new lesions were not applied.

Supplementary tables

Table S1: Baseline models for first-follow-up response.

A. Internal cohort

Model	Internal AUC	Internal adjusted p	Internal sensitivity / specificity
M0: Clinical	0.55 [0.44–0.65]	1.000	0.78 / 0.39
M1: Volumetric	0.54 [0.47–0.62]	—	0.43 / 0.66
M2: Radiomics	0.59 [0.54–0.64]	1.000	0.56 / 0.59
M3: Volumetric + Radiomics	0.58 [0.53–0.63]	1.000	0.31 / 0.83
M4: Comprehensive	0.58 [0.53–0.64]	1.000	0.29 / 0.88

B. External cohort

Model	External AUC	External adjusted p	External sensitivity / specificity
M0: Clinical	0.63 [0.49–0.81]	1.000	0.95 / 0.03
M1: Volumetric	0.54 [0.35–0.70]	—	0.50 / 0.52
M2: Radiomics	0.48 [0.37–0.62]	1.000	0.14 / 0.82
M3: Volumetric + Radiomics	0.58 [0.50–0.71]	1.000	0.04 / 1.00
M4: Comprehensive	0.58 [0.48–0.70]	1.000	0.03 / 1.00

Table S2: CT acquisition parameters (external values from an illustrative subset).

**Parameter**	**Internal**	**External**
Scanner manufacturer and model	Siemens SOMATOM Definition Flash	Siemens: Biograph 40, Emotion 16, NAEOTOM Alpha, SOMATOM Definition series, SOMATOM Force, SOMATOM go.Up, Sensation 64
Tube voltage	Sn140 kV / 100 kVp	90–140 kVp
In-plane resolution (mm)	0.65–0.98	0.35–1.17
Slice thickness (mm)	0.65–3.05	1.0–5.0
Reconstruction kernel	B31f, I31f, B40f, B41s	B30/31/70, Bl57/60, Br36/38/40/60, I30/31/70
Contrast phase	Arterial thoracic and portal-venous abdominal series, with some descriptions unspecified	Arterial thoracic, portal-venous abdominal, non-contrast, and contrast-enhanced series with unspecified phase
Radiomics discretization	MintLesion, fixed bin size 5 HU	MintLesion, fixed bin size 5 HU
Resampling	1 x 1 x 1 mm	1 x 1 x 1 mm
HU resegmentation range	-1024 to 2048	-1024 to 2048
GLCM aggregation	3D, averaged over directions, distance 1	3D, averaged over directions, distance 1

Abbreviations: 3D = three-dimensional. DICOM = Digital Imaging and Communications in Medicine. kV = kilovolt. kVp = kilovolt peak. Sn = tin-filtered spectrum.

Table S3: Radiomic feature classes.

Feature class	Features per timepoint, n	FU2 predictors, n	Description and representative measures	Discretization
Intensity-based statistical	21	84	Attenuation level, spread and distribution shape. Examples include percentiles, variance, skewness, kurtosis, energy and root mean square.	None (raw HU)
Intensity histogram	25	100	Distribution of discretized gray levels. Examples include entropy, uniformity, moments and histogram gradients.	Fixed 5 HU bins
Gray-level co-occurrence matrix (GLCM)	26	104	Local relationships between neighboring gray levels. Examples include contrast, dissimilarity, correlation, inverse difference and joint entropy.	Fixed 5 HU bins
Total	72	288		

Counts refer to the MintLesion features exported for this study before feature selection. FU2 predictors comprise baseline and first-follow-up values and their absolute and relative changes. The baseline-only analysis uses 72 radiomic predictors. Shape descriptors are not part of the primary radiomic set. HU = Hounsfield unit. FU2 = second follow-up.

Table S4: Feature-extraction settings and software versions.

A. Exports

Cohort	Study arm	Rows in export, n	Response criteria
Internal	Abdominal organs	6650	RECIST 1.1 (pre 3.11)
Internal	Abdominal lesions	5498	RECIST 1.1 (pre 3.11)
Internal	Dual-energy abdomen	8934	RECIST 1.1 (pre 3.11)
Internal	Dual-energy iodine	10572	RECIST 1.1 (pre 3.11)
Internal	recist	18	RECIST 1.1
Internal	Thoracic lesions	6104	RECIST 1.1 (pre 3.11)
External	Lesion tracking	1166	Manuell

B. Settings shared by all exports

Parameter	Value
Resampling filter	1
Resample spacing X (mm)	1
Resample spacing Y (mm)	1
Resample spacing Z (mm)	1
Discretisation method	FBS
Bin width (HU)	5
Laplacian-of-Gaussian filter	0
LoG sigma	1
GLCM distance	1
GLCM aggregation	3D average directions (3D:avg)
Intensity threshold filter	1
Threshold minimum (HU)	-1024
Threshold maximum (HU)	2048

C. Software versions

Component	Version
Python	3.12.13
platform	Linux-6.17.0-41-generic-x86_64-with-glibc2.42
numpy	2.3.5
pandas	2.3.3
scipy	1.16.1
scikit-learn	1.8.0
SimpleITK	2.5.3
PyRadiomics	3.1.0a2.post8+g6a761c4
XGBoost	3.4.0
SHAP	0.52.0
statsmodels	0.14.6
neuroCombat	0.2.12
MintLesion	13.11

Table S5: Radiomic feature distributions across cohorts.

**Parameter**	**Histogram/GLCM (discretized)**	**Intensity (raw HU)**
Features, n	204	84
Median |SMD| between cohorts	0.139	0.122
Maximum |SMD| between cohorts	0.974	0.739
Features with |SMD| > 0.8, %	1.0%	0.0%
Median Kolmogorov–Smirnov statistic	0.147	0.162
Features with Kolmogorov–Smirnov p < 0.05, %	78.9%	81.0%

Abbreviations: SMD = standardised mean difference.

Table S6: Model specifications.

A. Feature counts: follow-up 2

Parameter	M0	M1	M2	M3	M4
Candidate features, n	11	3	288	291	302
Clinical features, n	7	0	0	0	7
Treatment indicators, n	4	0	0	0	4
Volumetric features, n	0	3	0	3	3
Radiomic features, n	0	0	288	288	288

B. Feature counts: follow-up 1

Parameter	M0	M1	M2	M3	M4
Candidate features, n	10	2	72	74	84
Clinical features, n	6	0	0	0	6
Treatment indicators, n	4	0	0	0	4
Volumetric features, n	0	2	0	2	2
Radiomic features, n	0	0	72	72	72

C. Feature composition

Endpoint	Model	Feature list
Follow-up 2	M0: Clinical	Age<br>Sex<br>Affected organs, baseline<br>Affected organs, follow-up 1<br>Time since stage IV diagnosis<br>Time since first diagnosis<br>LDH, baseline<br>Treatment: ipilimumab/nivolumab<br>Treatment: other<br>Treatment: nivolumab<br>Treatment: pembrolizumab
Follow-up 2	M1: Volumetric	Log volume, baseline<br>Log volume, follow-up 1<br>Baseline to follow-up 1 interval
Follow-up 2	M2: Radiomics	GLCM angular second moment, baseline<br>GLCM angular second moment, follow-up 1<br>GLCM auto correlation, baseline<br>GLCM auto correlation, follow-up 1<br>GLCM cluster prominence, baseline<br>GLCM cluster prominence, follow-up 1<br>GLCM cluster shade, baseline<br>GLCM cluster shade, follow-up 1<br>GLCM cluster tendency, baseline<br>GLCM cluster tendency, follow-up 1<br>GLCM contrast, baseline<br>GLCM contrast, follow-up 1<br>... (+276 more)

Continued on next page

Table S6 (continued)

**Endpoint**	**Model**	**Feature list**
Follow-up 2	M3: Volumetric + Radiomics	Log volume, baseline<br>Log volume, follow-up 1<br>Baseline to follow-up 1 interval<br>GLCM angular second moment, baseline<br>GLCM angular second moment, follow-up 1<br>GLCM auto correlation, baseline<br>GLCM auto correlation, follow-up 1<br>GLCM cluster prominence, baseline<br>GLCM cluster prominence, follow-up 1<br>GLCM cluster shade, baseline<br>GLCM cluster shade, follow-up 1<br>GLCM cluster tendency, baseline<br>... (+279 more)
Follow-up 2	M4: Comprehensive	Age<br>Sex<br>Affected organs, baseline<br>Affected organs, follow-up 1<br>Time since stage IV diagnosis<br>Time since first diagnosis<br>LDH, baseline<br>Treatment: ipilimumab/nivolumab<br>Treatment: other<br>Treatment: nivolumab<br>Treatment: pembrolizumab<br>Log volume, baseline<br>... (+290 more)
Follow-up 1	M0: Clinical	Age<br>Sex<br>Affected organs, baseline<br>Time since stage IV diagnosis<br>Time since first diagnosis<br>LDH, baseline<br>Treatment: ipilimumab/nivolumab<br>Treatment: other<br>Treatment: nivolumab<br>Treatment: pembrolizumab
Follow-up 1	M1: Volumetric	Log volume, baseline<br>Baseline to follow-up 1 interval
Follow-up 1	M2: Radiomics	GLCM angular second moment, baseline<br>GLCM auto correlation, baseline<br>GLCM cluster prominence, baseline<br>GLCM cluster shade, baseline<br>GLCM cluster tendency, baseline<br>GLCM contrast, baseline<br>GLCM correlation, baseline<br>GLCM difference average, baseline<br>GLCM difference entropy, baseline<br>GLCM difference variance, baseline<br>GLCM dissimilarity, baseline<br>GLCM information correlation 1, baseline<br>... (+60 more)

Continued on next page

Table S6 (continued)

**Endpoint**	**Model**	**Feature list**
Follow-up 1	M3: Volumetric + Radiomics	Log volume, baseline<br>Baseline to follow-up 1 interval<br>GLCM angular second moment, baseline<br>GLCM auto correlation, baseline<br>GLCM cluster prominence, baseline<br>GLCM cluster shade, baseline<br>GLCM cluster tendency, baseline<br>GLCM contrast, baseline<br>GLCM correlation, baseline<br>GLCM difference average, baseline<br>GLCM difference entropy, baseline<br>GLCM difference variance, baseline<br>... (+62 more)
Follow-up 1	M4: Comprehensive	Age<br>Sex<br>Affected organs, baseline<br>Time since stage IV diagnosis<br>Time since first diagnosis<br>LDH, baseline<br>Treatment: ipilimumab/nivolumab<br>Treatment: other<br>Treatment: nivolumab<br>Treatment: pembrolizumab<br>Log volume, baseline<br>Baseline to follow-up 1 interval<br>... (+72 more)

Table S7: Internal and external cohort characteristics.

**Parameter**	**Internal**	**External**	**Test**	**Test statistic**	**p**
Age (years)	62.2 (12.6)	64.4 (13.4)	Welch t test	-0.83	0.410
Sex, n male (%)	60 (46.5)	18 (62.1)	Chi-square test (df = 1)	1.71	0.191
ICI regimen, n	Ipilimumab/nivolumab 51, Nivolumab 34, Pembrolizumab 44, Other 0	Ipilimumab/nivolumab 26, Nivolumab 1, Pembrolizumab 1, Other 1	Chi-square test (df = 3)	30.08	< 0.001
Affected organs at baseline	2 (1, 10)	3 (1, 6)	Mann–Whitney U test	1823.5	0.831
Baseline–FU1 interval (days)	95 (38, 174)	92 (51, 175)	Mann–Whitney U test	1943.5	0.745
FU1–FU2 interval (days)	89 (37, 618)	91 (12, 120)	Mann–Whitney U test	1295.5	0.506
LDH at baseline (U/L), n = 126/29	248 (134, 1451)	264 (146, 625)	Mann–Whitney U test	1653	0.426
Baseline lesion volume (mL)	0.90 [0.32-2.96]	0.24 [0.06-1.70]	Mann–Whitney U test	252756.5	< 0.001
Lesion organ distribution, % of lesions	Liver 29%, Lung 24%, Lymph nodes 19%, Skin 9%, Other 18%	Liver 14%, Lung 29%, Lymph nodes 24%, Skin 21%, Other 12%	Chi-square test (df = 4)	54.86	< 0.001
Clinical benefit at FU1, n (%) of lesions	991 (73.5%)	243 (87.4%)	Chi-square test (df = 1)	23.56	< 0.001
Clinical benefit at FU2, n (%) of lesions	855 (80.2%)	163 (93.1%)	Chi-square test (df = 1)	16.2	< 0.001

Table S8: Model performance metrics.

A. Internal cohort

Parameter	M0	M1	M2	M3	M4
Lesions, n (patients)	1052 (107)	1052 (107)	1052 (107)	1052 (107)	1052 (107)
Progression events, n	206	206	206	206	206
AUC	0.62 [0.48–0.73]	0.84 [0.78–0.88]	0.86 [0.81–0.90]	0.85 [0.80–0.89]	0.87 [0.82–0.91]
PR-AUC	0.32 [0.16–0.50]	0.47 [0.32–0.63]	0.52 [0.40–0.64]	0.51 [0.39–0.63]	0.54 [0.41–0.69]
PR-AUC prevalence baseline	0.196	0.196	0.196	0.196	0.196
Brier score	0.156	0.127	0.118	0.12	0.116
Balanced accuracy	0.623	0.783	0.809	0.805	0.813
Sensitivity	0.771	0.735	0.748	0.794	0.727
Specificity	0.476	0.83	0.869	0.816	0.898
PPV	0.858	0.947	0.959	0.946	0.967
NPV	0.336	0.433	0.457	0.491	0.445
True positives, n	652	622	633	672	615
False positives, n	108	35	27	38	21
True negatives, n	98	171	179	168	185
False negatives, n	194	224	213	174	231
Calibration intercept	0.076	-0.034	0.035	0.041	0.052
Calibration slope	0.399	0.632	0.59	0.509	0.703
Operating threshold	0.728	0.84	0.82	0.765	0.86

B. External cohort

Parameter	M0	M1	M2	M3	M4
Lesions, n (patients)	148 (20)	148 (20)	148 (20)	148 (20)	148 (20)
Progression events, n	12	12	12	12	12
AUC	0.69 [0.48–0.91]	0.68 [0.50–0.85]	0.68 [0.51–0.88]	0.69 [0.51–0.88]	0.70 [0.53–0.89]
PR-AUC	0.16 [0.06–0.68]	0.17 [0.06–0.34]	0.32 [0.07–0.60]	0.26 [0.07–0.47]	0.33 [0.08–0.60]
PR-AUC prevalence baseline	0.081	0.081	0.081	0.081	0.081
Brier score	0.088	0.107	0.105	0.105	0.101
Balanced accuracy	0.63	0.588	0.597	0.543	0.624
Sensitivity	0.926	0.676	0.61	0.669	0.581
Specificity	0.333	0.5	0.583	0.417	0.667
PPV	0.94	0.939	0.943	0.929	0.952
NPV	0.286	0.12	0.117	0.1	0.123
True positives, n	126	92	83	91	79
False positives, n	8	6	5	7	4
True negatives, n	4	6	7	5	8
False negatives, n	10	44	53	45	57
Calibration intercept	1.112	0.96	1.279	1.141	1.177
Calibration slope	1.341	0.237	0.351	0.263	0.324
Operating threshold	0.728	0.84	0.82	0.765	0.86

Table S9: Model comparisons and equivalence.

A. Follow-up 2, internal cohort

Model	ΔAUC vs M1	p	Adjusted p	Within ±0.05 margin
M0: Clinical	-0.212 [-0.357, -0.100]	< 0.001	< 0.001	No
M2: Radiomics	+0.020 [-0.013, +0.056]	0.228	0.456	No
M3: Volumetric + Radiomics	+0.015 [-0.016, +0.050]	0.356	0.456	Yes
M4: Comprehensive	+0.031 [+0.001, +0.066]	0.047	0.141	No

B. Follow-up 2, external cohort

Model	ΔAUC vs M1	p	Adjusted p	Within ±0.05 margin
M0: Clinical	+0.010 [-0.275, +0.329]	0.949	1.000	No
M2: Radiomics	+0.000 [-0.055, +0.081]	0.876	1.000	No
M3: Volumetric + Radiomics	+0.002 [-0.053, +0.084]	0.832	1.000	No
M4: Comprehensive	+0.018 [-0.038, +0.102]	0.458	1.000	No

C. Follow-up 1, internal cohort

Model	ΔAUC vs M1	p	Adjusted p	Within ±0.05 margin
M0: Clinical	+0.008 [-0.146, +0.146]	0.921	1.000	No
M2: Radiomics	+0.045 [-0.049, +0.145]	0.359	1.000	No
M3: Volumetric + Radiomics	+0.036 [-0.055, +0.132]	0.447	1.000	No
M4: Comprehensive	+0.042 [-0.060, +0.149]	0.415	1.000	No

D. Follow-up 1, external cohort

Model	ΔAUC vs M1	p	Adjusted p	Within ±0.05 margin
M0: Clinical	+0.091 [-0.147, +0.399]	0.441	1.000	No
M2: Radiomics	-0.062 [-0.264, +0.172]	0.647	1.000	No
M3: Volumetric + Radiomics	+0.041 [-0.105, +0.217]	0.548	1.000	No
M4: Comprehensive	+0.040 [-0.144, +0.293]	0.645	1.000	No

Table S10: Feature selection and importance.

A. Selection across folds

Model	Feature	Folds selected in, n	Folds selected in, %
M0: Clinical	Age	4	100%
M0: Clinical	Sex	4	100%
M0: Clinical	Affected organs, baseline	4	100%
M0: Clinical	Affected organs, follow-up 1	4	100%
M0: Clinical	Time since stage IV diagnosis	4	100%
M0: Clinical	Time since first diagnosis	4	100%
M0: Clinical	LDH, baseline	4	100%
M0: Clinical	Treatment: ipilimumab/nivolumab	4	100%
M0: Clinical	Treatment: nivolumab	4	100%
M0: Clinical	Treatment: pembrolizumab	4	100%
M1: Volumetric	Log volume, baseline	4	100%
M1: Volumetric	Log volume, follow-up 1	4	100%
M1: Volumetric	Baseline to follow-up 1 interval	4	100%
M2: Radiomics	GLCM information correlation 1, follow-up 1	4	100%
M2: Radiomics	GLCM inverse variance, follow-up 1	4	100%
M2: Radiomics	Histogram minimum histogram gradient, relative Δ	4	100%
M2: Radiomics	Histogram maximum histogram gradient, relative Δ	3	75%
M2: Radiomics	GLCM inverse difference moment normalized, follow-up 1	3	75%
M2: Radiomics	GLCM inverse difference normalized, relative Δ	3	75%
M2: Radiomics	GLCM inverse difference, follow-up 1	2	50%
M2: Radiomics	GLCM inverse difference moment, follow-up 1	2	50%
M2: Radiomics	Intensity 90th percentile, follow-up 1	2	50%
M2: Radiomics	Intensity max, follow-up 1	2	50%
M2: Radiomics	GLCM inverse difference moment normalized, relative Δ	2	50%
M2: Radiomics	Intensity min, follow-up 1	1	25%
M2: Radiomics	GLCM inverse difference moment, Δ	1	25%
M2: Radiomics	GLCM inverse difference normalized, follow-up 1	1	25%
M2: Radiomics	Intensity 50th percentile, follow-up 1	1	25%
M2: Radiomics	Intensity mean, follow-up 1	1	25%
M2: Radiomics	Histogram median abs deviation, baseline	1	25%
M2: Radiomics	Intensity median absolute deviation, relative Δ	1	25%
M2: Radiomics	Intensity 75th percentile, follow-up 1	1	25%
M2: Radiomics	GLCM inverse difference normalized, Δ	1	25%
M3: Volumetric + Radiomics	Log volume, follow-up 1	4	100%
M3: Volumetric + Radiomics	Histogram minimum histogram gradient, relative Δ	4	100%
M3: Volumetric + Radiomics	GLCM information correlation 1, follow-up 1	3	75%
M3: Volumetric + Radiomics	GLCM inverse variance, follow-up 1	3	75%
M3: Volumetric + Radiomics	Histogram maximum histogram gradient, relative Δ	3	75%

Continued on next page

Table S10 (continued)

Model	Feature	Folds selected in, n	Folds selected in, %
M3: Volumetric + Radiomics	GLCM inverse difference normalized, relative Δ	3	75%
M3: Volumetric + Radiomics	GLCM inverse difference, follow-up 1	2	50%
M3: Volumetric + Radiomics	Intensity 50th percentile, follow-up 1	2	50%
M3: Volumetric + Radiomics	GLCM inverse difference moment normalized, follow-up 1	2	50%
M3: Volumetric + Radiomics	Intensity 75th percentile, follow-up 1	2	50%
M3: Volumetric + Radiomics	Intensity min, follow-up 1	1	25%
M3: Volumetric + Radiomics	Intensity root mean square, baseline	1	25%
M3: Volumetric + Radiomics	GLCM inverse difference moment, Δ	1	25%
M3: Volumetric + Radiomics	GLCM inverse difference moment, follow-up 1	1	25%
M3: Volumetric + Radiomics	GLCM inverse difference normalized, follow-up 1	1	25%
M3: Volumetric + Radiomics	Intensity mean, follow-up 1	1	25%
M3: Volumetric + Radiomics	Histogram median abs deviation, baseline	1	25%
M3: Volumetric + Radiomics	Intensity 25th percentile, follow-up 1	1	25%
M3: Volumetric + Radiomics	Intensity median absolute deviation, relative Δ	1	25%
M3: Volumetric + Radiomics	Intensity 90th percentile, follow-up 1	1	25%
M3: Volumetric + Radiomics	GLCM inverse difference moment normalized, relative Δ	1	25%
M3: Volumetric + Radiomics	GLCM inverse difference normalized, Δ	1	25%
M4: Comprehensive	Log volume, follow-up 1	4	100%
M4: Comprehensive	Histogram minimum histogram gradient, relative Δ	4	100%
M4: Comprehensive	GLCM information correlation 1, follow-up 1	3	75%
M4: Comprehensive	GLCM inverse difference moment, follow-up 1	3	75%
M4: Comprehensive	Histogram maximum histogram gradient, relative Δ	3	75%
M4: Comprehensive	Affected organs, follow-up 1	2	50%
M4: Comprehensive	LDH, baseline	2	50%
M4: Comprehensive	GLCM inverse difference, follow-up 1	2	50%
M4: Comprehensive	GLCM inverse difference normalized, follow-up 1	2	50%
M4: Comprehensive	Affected organs, baseline	1	25%
M4: Comprehensive	Intensity 90th percentile, follow-up 1	1	25%
M4: Comprehensive	Intensity root mean square, baseline	1	25%
M4: Comprehensive	GLCM inverse difference moment, Δ	1	25%
M4: Comprehensive	GLCM inverse difference moment normalized, follow-up 1	1	25%
M4: Comprehensive	Intensity 50th percentile, follow-up 1	1	25%

Continued on next page

Table S10 (continued)

**Model**	**Feature**	**Folds selected in, n**	**Folds selected in, %**
M4: Comprehensive	Intensity mean absolute deviation, baseline	1	25%
M4: Comprehensive	GLCM joint entropy, Δ	1	25%
M4: Comprehensive	Intensity energy, Δ	1	25%
M4: Comprehensive	Intensity median absolute deviation, Δ	1	25%
M4: Comprehensive	GLCM inverse variance, follow-up 1	1	25%
M4: Comprehensive	Histogram sd, baseline	1	25%
M4: Comprehensive	Intensity mean, follow-up 1	1	25%
M4: Comprehensive	GLCM inverse difference normalized, Δ	1	25%
M4: Comprehensive	Intensity median absolute deviation, relative Δ	1	25%

B. Importance in the final combined model

Model	Feature	Feature class	Permutation importance (decrease in AUC)	Permutation importance, SD	Impurity importance
M3: Volumetric + Radiomics (final refit)	Histogram minimum histogram gradient, relative Δ	First-order (discretized)	0.0099	0.002	0.1695
M3: Volumetric + Radiomics (final refit)	Log volume, follow-up 1	Volumetric	0.0079	0.0015	0.1085
M3: Volumetric + Radiomics (final refit)	GLCM information correlation 1, follow-up 1	Texture (GLCM)	0.007	0.0011	0.0871
M3: Volumetric + Radiomics (final refit)	Histogram maximum histogram gradient, relative Δ	First-order (discretized)	0.004	0.0011	0.1668
M3: Volumetric + Radiomics (final refit)	Intensity 90th percentile, follow-up 1	First-order (raw HU)	0.0027	0.0006	0.083
M3: Volumetric + Radiomics (final refit)	GLCM inverse difference normalized, follow-up 1	Texture (GLCM)	0.0009	0.0002	0.0747
M3: Volumetric + Radiomics (final refit)	GLCM inverse variance, follow-up 1	Texture (GLCM)	0.0003	0.0001	0.0877
M3: Volumetric + Radiomics (final refit)	GLCM inverse difference normalized, relative Δ	Texture (GLCM)	0.0002	0.0001	0.0746
M3: Volumetric + Radiomics (final refit)	GLCM inverse difference normalized, Δ	Texture (GLCM)	0.0002	0.0001	0.0753
M3: Volumetric + Radiomics (final refit)	GLCM inverse difference moment, follow-up 1	Texture (GLCM)	0	0	0.0728

Table S11: Volume and redundancy sensitivity analyses.

Analysis	Lesions, n	Features, n	AUC	ΔAUC vs M1	p
M1: Volumetric (reference)	1066	3	0.84 [0.78–0.88]	reference	—
M2: Radiomics, raw	1053	288	0.86 [0.81–0.90]	+0.020 [-0.009, +0.058]	0.180
M3: Volumetric + Radiomics, raw	1053	291	0.85 [0.80–0.89]	+0.015 [-0.014, +0.053]	0.302
M2r: Radiomics, volume-residualized	1053	288	0.84 [0.78–0.89]	+0.005 [-0.031, +0.041]	0.712
M3r: Volumetric + residualized radiomics	1053	291	0.85 [0.79–0.89]	+0.010 [-0.023, +0.046]	0.513
M2d: Radiomics, $\|\rho\| \leq 0.5$ only	1053	216	0.84 [0.79–0.89]	+0.004 [-0.035, +0.043]	0.802
M3d: Volumetric + decorrelated radiomics	1053	219	0.85 [0.80–0.89]	+0.015 [-0.014, +0.049]	0.307
M3f: Volumetric + redundancy-filtered radiomics	1053	97	0.85 [0.79–0.90]	+0.015 [-0.030, +0.061]	0.406

Table S12: Lesion-size subgroup analyses.

A. Sample sizes and lesion characteristics

Size stratum	Lesions, n (patients)	Progression events, n	Clinical benefit, n	Median baseline volume (mL)	Approximate voxels at native resolution, n	Median grey levels
Size < 0.5 mL	390 (86)	71	319	0.22	81	24
Size 0.5–1 mL	190 (64)	35	155	0.72	265	23.5
Size 1–2 mL	160 (67)	26	134	1.39	512	24
Size 2–5 mL	150 (66)	38	112	3.02	1113	28
Size $\geq$ 5 mL	176 (61)	41	135	12.46	4589	32
All lesions $\geq$ 1 mL	486 (94)	105	381			
All lesions $\geq$ 2 mL	326 (82)	79	247			
All lesions $\geq$ 5 mL	176 (61)	41	135			

B. Model comparisons

Size stratum	M1: Volumetric AUC	M3: Volumetric + Radiomics AUC	ΔAUC vs M1	p	Adjusted p
Size < 0.5 mL	0.82 [0.73–0.89]	0.84 [0.77–0.91]	+0.035 [-0.006, +0.084]	0.109	0.654
Size 0.5–1 mL	0.89 [0.79–0.94]	0.89 [0.80–0.95]	+0.005 [-0.038, +0.051]	0.826	0.891
Size 1–2 mL	0.91 [0.84–0.96]	0.89 [0.81–0.95]	-0.018 [-0.059, +0.017]	0.301	0.891
Size 2–5 mL	0.78 [0.67–0.88]	0.82 [0.72–0.90]	+0.034 [-0.037, +0.099]	0.297	0.891
Size $\geq$ 5 mL	0.55 [0.42–0.69]	0.64 [0.48–0.79]	+0.088 [+0.000, +0.191]	0.050	0.400
All lesions $\geq$ 1 mL	0.78 [0.70–0.87]	0.82 [0.73–0.91]	+0.037 [-0.011, +0.092]	0.126	0.654
All lesions $\geq$ 2 mL	0.70 [0.59–0.80]	0.76 [0.63–0.87]	+0.059 [-0.017, +0.143]	0.135	0.654
All lesions $\geq$ 5 mL	0.55 [0.42–0.69]	0.64 [0.48–0.79]	+0.088 [+0.000, +0.191]	0.050	0.400

Table S13: Harmonization and feature-distribution filtering.

A. Internal cohort

Arm	Model	Internal lesions, n	Internal AUC	Internal ΔAUC vs M1	Internal adjusted p
Unharmonized	M0: Clinical	1052	0.62 [0.48–0.73]	-0.212 [-0.357, -0.100]	< 0.001
Unharmonized	M1: Volumetric	1052	0.84 [0.78–0.88]	reference	—
Unharmonized	M2: Radiomics	1052	0.86 [0.81–0.90]	+0.020 [-0.013, +0.056]	0.456
Unharmonized	M3: Volumetric + Radiomics	1052	0.85 [0.80–0.89]	+0.015 [-0.016, +0.050]	0.456
Unharmonized	M4: Comprehensive	1052	0.87 [0.82–0.91]	+0.031 [+0.001, +0.066]	0.141
ComBat	M0: Clinical	1052	0.62 [0.48–0.73]	-0.212 [-0.357, -0.100]	< 0.001
ComBat	M1: Volumetric	1052	0.84 [0.78–0.88]	reference	—
ComBat	M2: Radiomics	1052	0.86 [0.81–0.90]	+0.021 [-0.010, +0.055]	0.336
ComBat	M3: Volumetric + Radiomics	1052	0.86 [0.81–0.90]	+0.021 [-0.008, +0.055]	0.336
ComBat	M4: Comprehensive	1052	0.87 [0.82–0.90]	+0.030 [-0.001, +0.064]	0.180
Stability-filtered	M0: Clinical	1052	0.62 [0.48–0.73]	-0.212 [-0.357, -0.100]	< 0.001
Stability-filtered	M1: Volumetric	1052	0.84 [0.78–0.88]	reference	—
Stability-filtered	M2: Radiomics	1052	0.86 [0.80–0.90]	+0.022 [-0.009, +0.055]	0.216
Stability-filtered	M3: Volumetric + Radiomics	1052	0.86 [0.81–0.90]	+0.026 [-0.004, +0.060]	0.216
Stability-filtered	M4: Comprehensive	1052	0.88 [0.83–0.92]	+0.041 [+0.012, +0.071]	0.012

B. External cohort

Arm	Model	External lesions, n	External progression events, n	External AUC	External ΔAUC vs M1	External adjusted p
Unharmonized	M0: Clinical	148	12	0.69 [0.48–0.91]	+0.010 [-0.275, +0.329]	1.000
Unharmonized	M1: Volumetric	148	12	0.68 [0.50–0.85]	reference	—
Unharmonized	M2: Radiomics	148	12	0.68 [0.51–0.88]	+0.000 [-0.055, +0.081]	1.000
Unharmonized	M3: Volumetric + Radiomics	148	12	0.69 [0.51–0.88]	+0.002 [-0.053, +0.084]	1.000
Unharmonized	M4: Comprehensive	148	12	0.70 [0.53–0.89]	+0.018 [-0.038, +0.102]	1.000

Continued on next page

Table S13 (continued)

Arm	Model	External lesions, n	External progression events, n	External AUC	External ΔAUC vs M1	External adjusted p
ComBat	M0: Clinical	148	12	0.69 [0.48–0.91]	+0.010 [-0.275, +0.329]	0.949
ComBat	M1: Volumetric	148	12	0.68 [0.50–0.85]	reference	—
ComBat	M2: Radiomics	148	12	0.61 [0.46–0.75]	-0.069 [-0.191, +0.062]	0.825
ComBat	M3: Volumetric + Radiomics	148	12	0.63 [0.48–0.79]	-0.058 [-0.139, +0.048]	0.825
ComBat	M4: Comprehensive	148	12	0.62 [0.43–0.81]	-0.063 [-0.140, +0.010]	0.340
Stability-filtered	M0: Clinical	148	12	0.69 [0.48–0.91]	+0.010 [-0.275, +0.329]	1.000
Stability-filtered	M1: Volumetric	148	12	0.68 [0.50–0.85]	reference	—
Stability-filtered	M2: Radiomics	148	12	0.76 [0.61–0.90]	+0.073 [+0.000, +0.177]	0.204
Stability-filtered	M3: Volumetric + Radiomics	148	12	0.72 [0.58–0.90]	+0.040 [-0.046, +0.170]	1.000
Stability-filtered	M4: Comprehensive	148	12	0.72 [0.55–0.90]	+0.038 [-0.060, +0.176]	1.000

Table S14: Extended radiomic feature sets.

**Feature configuration**	**Lesions, n (patients)**	**Features, n**	**AUC**	**ΔAUC vs M1**	**Adjusted p**
M1: Volumetric (reference)	1043 (107)	3	0.82 [0.75–0.87]	reference	—
M2: texture + first-order, bin width 5 HU	837 (101)	372	0.81 [0.74–0.87]	+0.007 [-0.037, +0.052]	1.000
M3: volumetric + radiomics, bin width 5 HU	837 (101)	375	0.81 [0.75–0.87]	+0.007 [-0.030, +0.048]	1.000
M2: texture + first-order, bin width 10 HU	841 (102)	372	0.81 [0.74–0.87]	+0.009 [-0.031, +0.055]	1.000
M3: volumetric + radiomics, bin width 10 HU	841 (102)	375	0.81 [0.75–0.87]	+0.012 [-0.027, +0.056]	1.000
M2: texture + first-order, bin width 25 HU	842 (102)	372	0.84 [0.79–0.88]	+0.037 [-0.006, +0.083]	0.900
M3: volumetric + radiomics, bin width 25 HU	842 (102)	375	0.84 [0.79–0.88]	+0.040 [+0.001, +0.084]	0.456
M2: texture + first-order, bin width 64 HU	840 (102)	372	0.83 [0.78–0.87]	+0.027 [-0.015, +0.069]	1.000
M3: volumetric + radiomics, bin width 64 HU	840 (102)	375	0.83 [0.77–0.87]	+0.026 [-0.018, +0.070]	1.000
M2: higher-order texture only	858 (102)	300	0.83 [0.78–0.87]	+0.031 [-0.008, +0.069]	1.000
M3: volumetric + higher-order texture	858 (102)	303	0.83 [0.78–0.88]	+0.036 [+0.001, +0.073]	0.480
M2: wavelet + LoG	807 (101)	4092	0.85 [0.79–0.89]	+0.051 [+0.009, +0.093]	0.168
M3: volumetric + wavelet + LoG	807 (101)	4095	0.85 [0.80–0.89]	+0.057 [+0.017, +0.101]	0.096
M2: full radiomics pipeline	792 (101)	4464	0.83 [0.77–0.88]	+0.033 [-0.010, +0.082]	1.000
M3: volumetric + full radiomics pipeline	792 (101)	4467	0.84 [0.78–0.88]	+0.041 [+0.001, +0.085]	0.456
M2: shape features alone	842 (101)	56	0.84 [0.79–0.89]	+0.041 [+0.008, +0.075]	0.150
M3: volumetric + shape	842 (101)	59	0.83 [0.78–0.88]	+0.033 [+0.004, +0.065]	0.364

Table S15: Follow-up attrition.

A. Lesion and patient flow

Parameter	Internal	External	Pooled
Follow-up 1 lesions, n	1348	278	1626
Follow-up 1 patients, n	129	29	158
Follow-up 2 lesions, n	1066	175	1241
Follow-up 2 patients, n	108	23	131
Lesions lost, n (%)	282 (20.9)	103 (37.1)	385 (23.7)
Patients lost, n	21	6	27

B. Recorded lesion state underlying each loss

Recorded lesion state at follow-up 2	Lesions, n (%)	Meaning
(no FU2 scan / not recorded)	384 (99.7)	no second follow-up scan
Vorhanden	1 (0.3)	present

C. First-follow-up response of lost versus retained lesions

Response at follow-up 1	Lesions lost, n (%)	Lesions retained, n (%)
CR	9 (2.3)	173 (13.9)
PD	124 (32.2)	268 (21.6)
PR	28 (7.3)	257 (20.7)
SD	224 (58.2)	543 (43.8)

Abbreviations: PD = progressive disease.

Table S16: Inter-observer agreement.

A. Agreement by feature family

Feature family	Bin width (HU)	Features, n	Median ICC(2,1)	ICC interquartile range	Features with ICC ≥ 0.90, %
First-order	25	18	0.98	0.932–0.988	78%
Shape / size	25	14	0.946	0.766–0.953	71%
Texture	25	75	0.948	0.795–0.970	67%
First-order	64	18	0.984	0.932–0.988	78%
Shape / size	64	14	0.946	0.766–0.953	71%
Texture	64	75	0.93	0.746–0.969	56%

B. Volume and diameter, per feature

Feature	Lesions, n	ICC(2,1)
shape_MeshVolume	41	0.953
shape_VoxelVolume	41	0.953
shape_SurfaceArea	41	0.946
shape_Maximum2DDiameterSlice	41	0.953
shape_Maximum2DDiameterColumn	41	0.947
shape_Maximum2DDiameterRow	41	0.913
shape_Maximum3DDiameter	41	0.37

Table S17: Radiomics Quality Score self-assessment.

Item	Criterion	Points awarded	Maximum	Justification
1	Image protocol quality	1	2	Internal protocols and an illustrative external DICOM subset are summarized in Supplementary Table S2. Protocols are not publicly available, and the external subset does not represent the complete cohort.
2	Multiple segmentations	1	1	Independent second reading of 41 corresponded lesions with per-feature ICC(2,1) (Supplementary Table S16).
3	Phantom study	0	1	No phantom study was performed.
4	Imaging at multiple time points	1	1	Baseline and first follow-up imaging enter every model and delta features are computed between them.
5	Feature reduction or adjustment for multiple testing	3	3	Variance filtering, univariate preselection and recursive feature elimination were performed within each training fold. Holm-Bonferroni adjustment was applied within every comparison family.
6	Multivariable analysis with non-radiomics features	1	1	Clinical, volumetric and combined models are compared throughout (M0 to M4).
7	Detect and discuss biological correlates	0	1	No biological or histological correlates were analyzed.
8	Cut-off analysis	1	1	Operating threshold fixed at the Youden point of the internal out-of-fold predictions and applied unchanged externally.
9	Discrimination statistics	2	2	AUC and precision-recall AUC with patient-level cluster-bootstrap confidence intervals for every model in both cohorts (Supplementary Table S8).
10	Calibration statistics	1	2	Calibration intercept, slope and curves are reported (Supplementary Table S8, Figure S1). Resampling-based uncertainty was not calculated for these statistics.
11	Prospective study registered in a trial database	0	7	Retrospective study.
12	Validation	3	5	External validation used a fully held-out cohort from one collaborating institution. No training information crossed cohorts.
13	Comparison to gold standard	2	2	All radiomics models are compared against the current size-based assessment (volume dynamics and a single-feature volume-change readout).
14	Potential clinical utility	2	2	Decision-curve analysis with net benefit against treat-all and treat-none strategies (Figure S1).
15	Cost-effectiveness analysis	0	1	Not performed.
16	Open science and data	0	4	Analysis code, de-identified feature matrices and extraction parameter files are available on request. Images cannot be shared. There is no open repository.
Total		18	36	18 of 36 points (50%).

Table S18: Patient-level sensitivity analysis.

Parameter	P0: Clinical	P1: Volumetric	P2: Radiomics	P3: Volumetric + Radiomics
Patients, n	107	108	108	108
Progression, n	23	23	23	23
Clinical benefit, n	84	85	85	85
AUC	0.44 [0.31–0.57]	0.89 [0.81–0.95]	0.87 [0.79–0.93]	0.87 [0.78–0.95]
ΔAUC vs P1	reference	reference	-0.024 [-0.075, +0.025]	-0.017 [-0.060, +0.026]
Adjusted p	—	—	0.664	0.664

Table S19: Excluding lesions that disappeared at first follow-up.

A. Internal cohort

Model	Lesions, n (disappeared at FU1)	AUC, all lesions	AUC, without disappeared lesions	ΔAUC vs M1, without disappeared lesions	p
M0: Clinical	1052 (133)	0.62 [0.48–0.73]	0.62 [0.48–0.73]	-0.183 [-0.338, -0.074]	< 0.001
M1: Volumetric	1052 (133)	0.84 [0.78–0.88]	0.81 [0.75–0.85]	reference	—
M2: Radiomics	1052 (133)	0.86 [0.81–0.90]	0.83 [0.78–0.88]	+0.023 [-0.013, +0.061]	0.219
M3: Volumetric + Radiomics	1052 (133)	0.85 [0.80–0.89]	0.82 [0.77–0.87]	+0.017 [-0.017, +0.056]	0.347
M4: Comprehensive	1052 (133)	0.87 [0.82–0.91]	0.84 [0.79–0.89]	+0.035 [+0.001, +0.074]	0.048

B. External cohort

Model	Lesions, n (disappeared at FU1)	AUC, all lesions	AUC, without disappeared lesions	ΔAUC vs M1, without disappeared lesions	p
M0: Clinical	148 (28)	0.69 [0.48–0.91]	0.66 [0.46–0.90]	+0.009 [-0.292, +0.392]	0.956
M1: Volumetric	148 (28)	0.68 [0.50–0.85]	0.65 [0.44–0.84]	reference	—
M2: Radiomics	148 (28)	0.68 [0.51–0.88]	0.60 [0.38–0.84]	-0.046 [-0.113, +0.041]	0.271
M3: Volumetric + Radiomics	148 (28)	0.69 [0.51–0.88]	0.60 [0.40–0.85]	-0.044 [-0.096, +0.039]	0.254
M4: Comprehensive	148 (28)	0.70 [0.53–0.89]	0.62 [0.42–0.86]	-0.023 [-0.079, +0.057]	0.639
